# Isolation and characterization of two novel phages with lytic activity against multidrug-resistant *Acinetobacter baumannii* strains: potential for phage therapy

**DOI:** 10.1038/s41598-025-27600-x

**Published:** 2025-12-10

**Authors:** Xi Chen, Zimeng Ma, Gege Feng, Zhirou Sun, Yating Zeng, Dan Li, Yingqi Qian, Qin Peng

**Affiliations:** Ministry of Education Key Laboratory for Ecology of Tropical Islands, College of Life Sciences, Hainan Normal University, Haikou, 571158 China

**Keywords:** *Acinetobacter baumannii*, Phage, Phage therapy, Microbiology, Molecular biology

## Abstract

The emergence of multidrug-resistant *Acinetobacter baumannii* (MDR-AB) has raised concerns regarding the lack of effective treatment options, prompting interest in phage therapy. In this study, two phages, designated vB_MZM_2AB-P and vB_MZM_4AB-P, were isolated and characterized for their lytic activity against MDR-AB strains. vB_MZM_2AB-P demonstrated considerable stability, retaining activity at 70 °C and pH 11.00, with over 37.10% survival rate after three months of storage. It displayed a 10-min latent period and a burst size of 39.72 PFU/cell. vB_MZM_4AB-P was stable at 50 °C and pH 7.00, but lost activity after three months of storage. Its latent period was also 10 min, with a burst size as high as 746.70 PFU/cell. Transmission electron microscopy revealed that phage vB_MZM_2AB-P has an icosahedral head with a diameter of 55.00 nm and a non-contractile tail that is 121.00 nm long, while phage vB_MZM_4AB-P has an icosahedral head with a diameter of 75.36 nm and a non-contractile tail that is 19.44 nm long. Genomic analysis showed that vB_MZM_2AB-P (43,664 bp) shared 94.18% and 92.80% similarity with phages DMU1 and SH-Ab15497, respectively, while vB_MZM_4AB-P (42,975 bp) shared 73.82% similarity with phage vB_AbaP_Acibel007. Phylogenetic analysis indicated that vB_MZM_2AB-P may represent a new genus within the order Caudoviricetes, and vB_MZM_4AB-P a new species within the genus *Daemvirus*. No antibiotic resistance or virulence genes were detected. These findings demonstrate that vB_MZM_2AB-P and vB_MZM_4AB-P are novel phages with distinct genomic features and favorable lytic activity against MDR-AB strains, highlighting their value for expanding phage diversity and for future fundamental research.

**Supplementary Information:**

The online version contains supplementary material available at 10.1038/s41598-025-27600-x.

## Introduction

*Acinetobacter baumannii*, a ubiquitous opportunistic pathogen in natural environments, is classified as one of the ESKAPE pathogens due to its multidrug resistance^1^. Carbapenem-resistant *A. baumannii* (CRAB) was designated as a “critical” priority pathogen by the World Health Organization (WHO) in 2008^2^. This bacterium exhibits rapid evolution of drug resistance, leading to the emergence of multidrug-resistant (MDR), extensively drug-resistant (XDR), and even pandrug-resistant (PDR) strains. According to the 2023 European Centre for Disease Prevention and Control (ECDC) report, the prevalence of MDR *A. baumannii* (MDR-AB) in Europe has reached alarming levels, with 74.50% of isolates resistant to at least one antibiotic, 66.60% resistant to fluoroquinolones, aminoglycosides, and carbapenems, and carbapenem resistance rates reaching 39.90%^3^. Currently, the increase in drug resistance has led to the significant prevalence of MDR-AB in hospital infections, with CRAB being a major concern^4^. Its resistance mechanisms include the production of β-lactamases and the integration of foreign DNA^5,6^, which further complicates treatment. In recent years, oxacillinases (OXAs) have become a prevalent mechanism of carbapenem resistance^7^. Notably, cefiderocol is considered a last-line treatment for CRAB strains, however its resistance rate has significantly increased to 8.80% in 2023^8^. More recent study with indicate cefiderocol susceptibility was only 60.7% in CRAB isolates^9^. The emergence of colistin-resistant *A. baumannii* further reduces the effectiveness of traditional treatments^10^. As a result, the efficacy of traditional drugs is facing severe challenges.

Phages, as viruses with high host specificity, have been shown to hold great potential as an alternative to traditional antibiotic treatments for bacterial infections^11^. Approximately 10^31^ phages exist on Earth, forming a massive natural antimicrobial drug reservoir that is widely distributed across various ecosystems and exhibits strong host specificity, avoiding negative impacts on commensal microbiota^12^. Research has shown that phages can efficiently inhibit drug-resistant bacteria at low doses^13^. Local phage therapy has achieved significant results in treating skin wound infections in diabetic mice and bacterial pneumonia with intranasal administration. In mouse models of bacteremia and wound infections, phage therapy exhibited significant inhibitory effects against *A. baumannii*^14^. Notably, during the COVID-19 pandemic, phage therapy aided the recovery of several patients infected with CRAB strains^15^.

In recent years, phage therapy has gained widespread attention for its application in treating antibiotic-resistant bacterial infections. To expand the phage lysis spectrum and delay the emergence of resistant strains, phage-antibiotic combination therapy (PAC) has been adopted. For example, Grygorcewicz et al*.* applied a phage cocktail combined with antibiotics in a urinary model, significantly reducing the biofilm formation by *A. baumannii*^16^, highlighting the potential of PAC in managing complex infections^17^.

Multidrug-resistant *A. baumannii* poses a critical challenge to current antimicrobial therapies, and novel bacteriophages offer a promising alternative. We hypothesized that lytic phages with potent activity and unique genomic features could be isolated to target MDR-AB effectively. This study aimed to discover and characterize such phages to expand the phage resource pool and provide a foundation for developing phage-based strategies, including cocktails or combination therapies, against MDR-AB infections.

## Results

### Phage morphology features

**Fig. 1:**
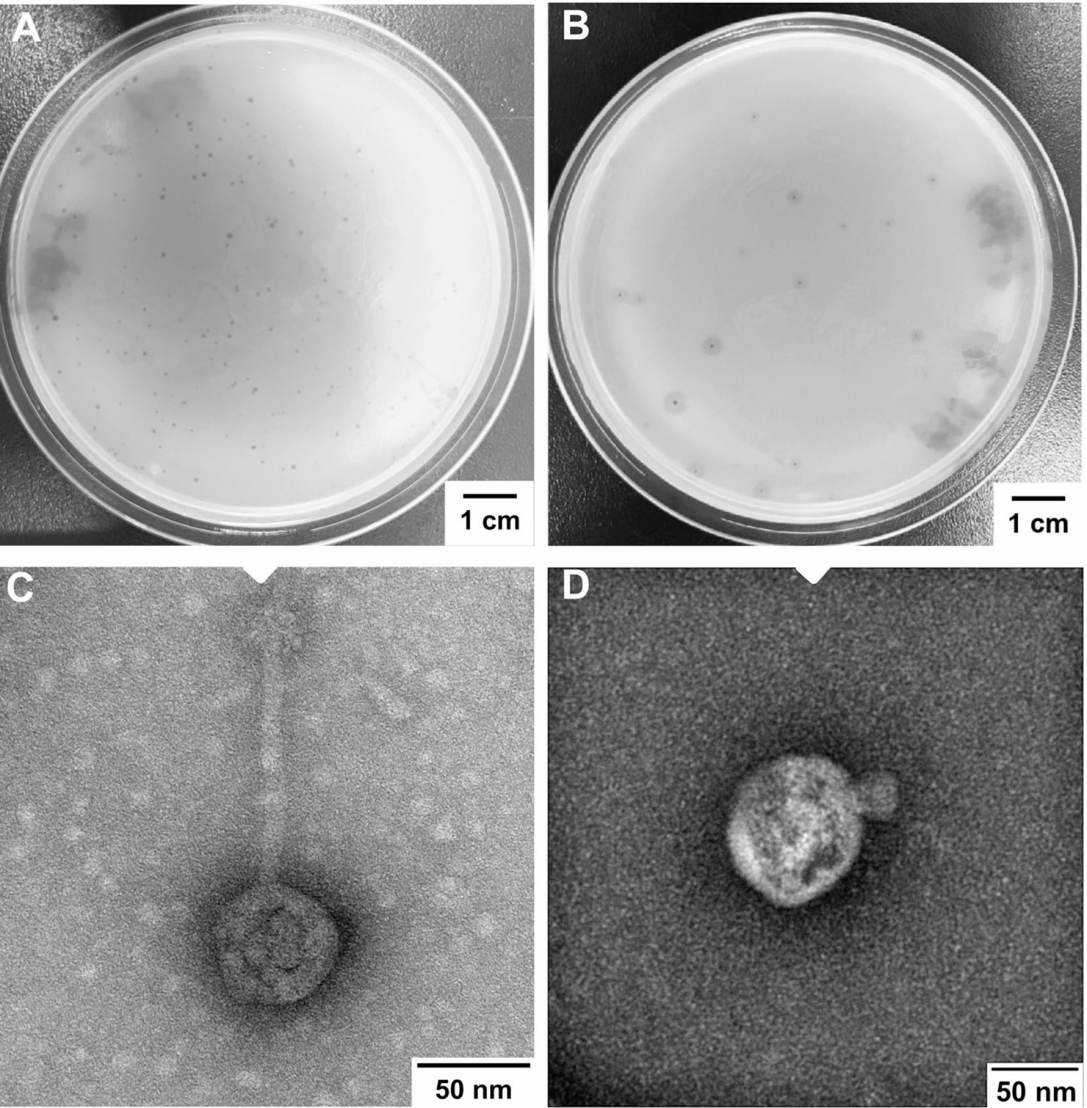
Morphological observation of phages vB_MZM_2AB-P and vB_MZM_4AB-P. Plaque images of phage vB_MZM_2AB-P (**A**) and vB_MZM_4AB-P (**B**), scale bar = 1.00 cm. TEM images of phage vB_MZM_2AB-P (**C**) and vB_MZM_4AB-P (**D**), scale bar = 50.00 nm. The distinct plaque formations and structural characteristics of the two phages are shown, highlighting their morphological differences.

Two phages were screened and purified using *A. baumannii* 2AB and 4AB as indicator strains and were designated vB_MZM_2AB-P and vB_MZM_4AB-P, respectively. These phages are deposited at the China Center for Type Culture Collection (CCTCC), with accession numbers CCTCC M 20,24,1199 (vB_MZM_2AB-P) and CCTCC M 20,24,1200 (vB_MZM_4AB-P). After 20 h of incubation, phage vB_MZM_2AB-P formed plaques on *A. baumannii* 2AB plates with a central diameter of 1.00 mm and no halo zone (Fig. 1A). In contrast, phage vB_MZM_4AB-P formed plaques on *A. baumannii* 4AB plates with a clear central diameter of 1.25 mm and a halo diameter of 2.10 mm (Fig. 1B). TEM analysis revealed that phage vB_MZM_2AB-P possesses an icosahedral head with a diameter of 55.00 nm and a noncontractile tail length of 121.00 nm (Fig. 1C), while phage vB_MZM_4AB-P has a head with a diameter of 75.36 nm and a 19.44 nm long noncontractile tail (Fig. 1D). Based on these morphological characteristics, vB_MZM_2AB-P and vB_MZM_4AB-P are classified as siphovirus-like and podovirus-like phages, respectively.

### Phenotypic antimicrobial susceptibility testing (AST) of *A. baumannii* isolates

The *A. baumannii* strains 2AB, 4AB, 6AB, and ATCC 17,978 exhibited resistance to most of the tested antibiotics, including penicillins, β-lactam combination agents, cephems, carbapenems, aminoglycosides, fluoroquinolones, and folate pathway antagonists (Figure S1). Strain 7AB also demonstrated resistance to penicillins, β-lactam combination agents, and faminoglycosides. Therefore, 2AB, 4AB, 6AB, ATCC 17,978, and 7AB are classified as multidrug-resistant strains. Among these, 2AB, 4AB, 6AB, and ATCC 17,978 exhibited resistance to imipenem and meropenem, categorizing them as carbapenem-resistant *A. baumannii* (CRAB) strains (Figure S1).

### Host range for the phages

Phages vB_MZM_2AB-P and vB_MZM_4AB-P exhibit host specificity. Both phages are capable of infecting multidrug-resistant strains and CRAB strains. Among the 24 bacterial strains tested, vB_MZM_2AB-P can only infect MDR *A. baumannii* 2AB and ATCC-19606, with lytic moderate activity against ATCC-19606 observed as a semi-confluent zone. While vB_MZM_4AB-P can infect MDR *A. baumannii* 2AB, 4AB, and 6AB (Table S1), and the lytic activity against strains 2AB and 6AB is also observed as a semi-confluent zone. When phages vB_MZM_2AB-P and vB_MZM_4AB-P were mixed in a 1:1 ratio, they could infect strains 2AB, 4AB, 6AB, and ATCC-19606. This combination effectively expanded and strictly covered the individual host range of each phage, demonstrating that their host targeting patterns are complementary, rather than redundant (Table S1). The experimental results indicate that the EOP of vB_MZM_4AB-P against 2AB is 0.0007, suggesting low lytic efficiency. No plaques were observed for phage vB_MZM_4AB-P on *A. baumannii* 6AB, nor for phage vB_MZM_2AB-P on *A. baumannii* ATCC-19606 during the EOP assay. Therefore, the lytic effects observed in the spot test for vB_MZM_4AB-P on *A. baumannii* 6AB and vB_MZM_2AB-P on *A. baumannii* ATCC-19606 may be attributed to lysis from without rather than efficient phage replication.

### Determination of the optimal MOI for phage infection

**Fig. 2:**
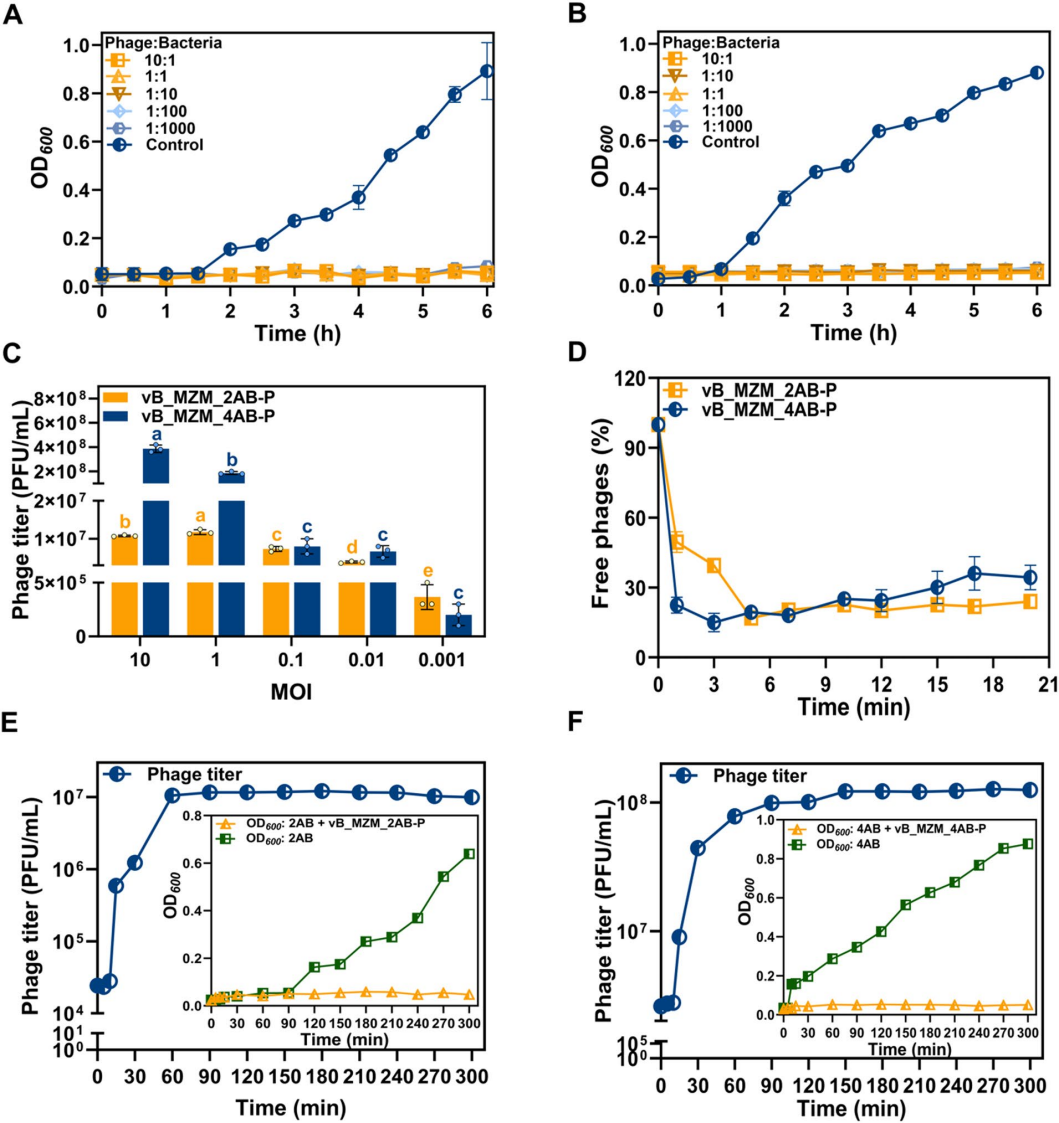
Infection dynamics analysis of phages vB_MZM_2AB-P and vB_MZM_4AB-P. Determine the optimal MOI value for phages vB_MZM_2AB-P (**A**) and vB_MZM_4AB-P (**B**). The phages are mixed with bacteria at different MOI values (MOI = 10:1, 1:1, 1:10, 1:100, 1:1000), and the control group is not treated with phages. (**C**) The effect of different MOI values on the growth of vB_MZM_2AB-P and vB_MZM_4AB-P after 6 h of incubation. (**D**) Adsorption curves of vB_MZM_2AB-P and vB_MZM_4AB-P to their host strains. One-step growth curves of phages vB_MZM_2AB-P (**E**) and vB_MZM_4AB-P (**F**), with the data represented as averages and error bars showing standard deviation (SD). The data are from three independent experiments. Different letters indicate significant differences (*p* < 0.05). The letters in orange and blue represent phage vB_MZM_2AB-P and vB_MZM_4AB-P, respectively. The most effective MOI for optimal bacterial growth suppression by both phages is identified, with the adsorption and growth dynamics provided.

To explore the infection efficiency of the phages, the optimal MOI for two phages was determined. Under the conditions of MOI = 10, 1, 0.1, 0.01, 0.001, the phage treatment groups all showed effective bacterial growth inhibition effects (Fig. 2A, B). When the MOI was 1, phage vB_MZM_2AB-P demonstrated the highest titer, reaching 1.18 × 10^7^ PFU/mL (*p* < 0.05), indicating that an MOI of 1 is the optimal MOI for vB_MZM_2AB-P (Fig. 2C). When the MOI was 10, the phage vB_MZM_4AB-P exhibited the highest titer of 3.80 × 10^8^ PFU/mL (*p* < 0.05), thus an MOI of 10 was identified as the optimal MOI for vB_MZM_4AB-P (Fig. 2C).The bacterial growth inhibition effects were significant for both phages, and even at an MOI of 0.001 (Fig. 2A, B), they effectively suppressed the growth of the host, indicating antibacterial activity. In terms of phage yield, both phages demonstrated substantial titers across a range of MOIs (Fig. 2C), suggesting infectivity.

### Determination of phage adsorption rate

To reveal the adsorption characteristics of the two phages with host cells, the adsorption rates of both phages were measured. The adsorption efficiency was assessed by detecting the residual phage content in the mixture after adsorption. The results showed that when phage vB_MZM_2AB-P was adsorbed for 5 min, the residual phage content was the lowest, with an adsorption rate of 83.10%. When phage vB_MZM_4AB-P was adsorbed for 3 min, the residual phage content was the lowest, with an adsorption rate of 85.00% (Fig. 2D). Both phages exhibited high adsorption rates toward their host cells.

### One-step growth of phage

To further investigate the infection characteristics of these two phages, a one-step growth curves were performed. The results indicated that the latent period of phage vB_MZM_2AB-P was 10 min, the lytic period lasted approximately 50 min, with a maximum titer of 1.12 × 10^7^ PFU/mL and a burst size of 39.72 PFU/cell (Fig. 2E). The latent period of phage vB_MZM_4AB-P was 10 min, and the lytic period lasted about 80 min, with a maximum titer of 1.27 × 10^8^ PFU/mL and a burst size of 746.70 PFU/cell (Fig. 2F).

### Phage stability assessment

**Fig. 3:**
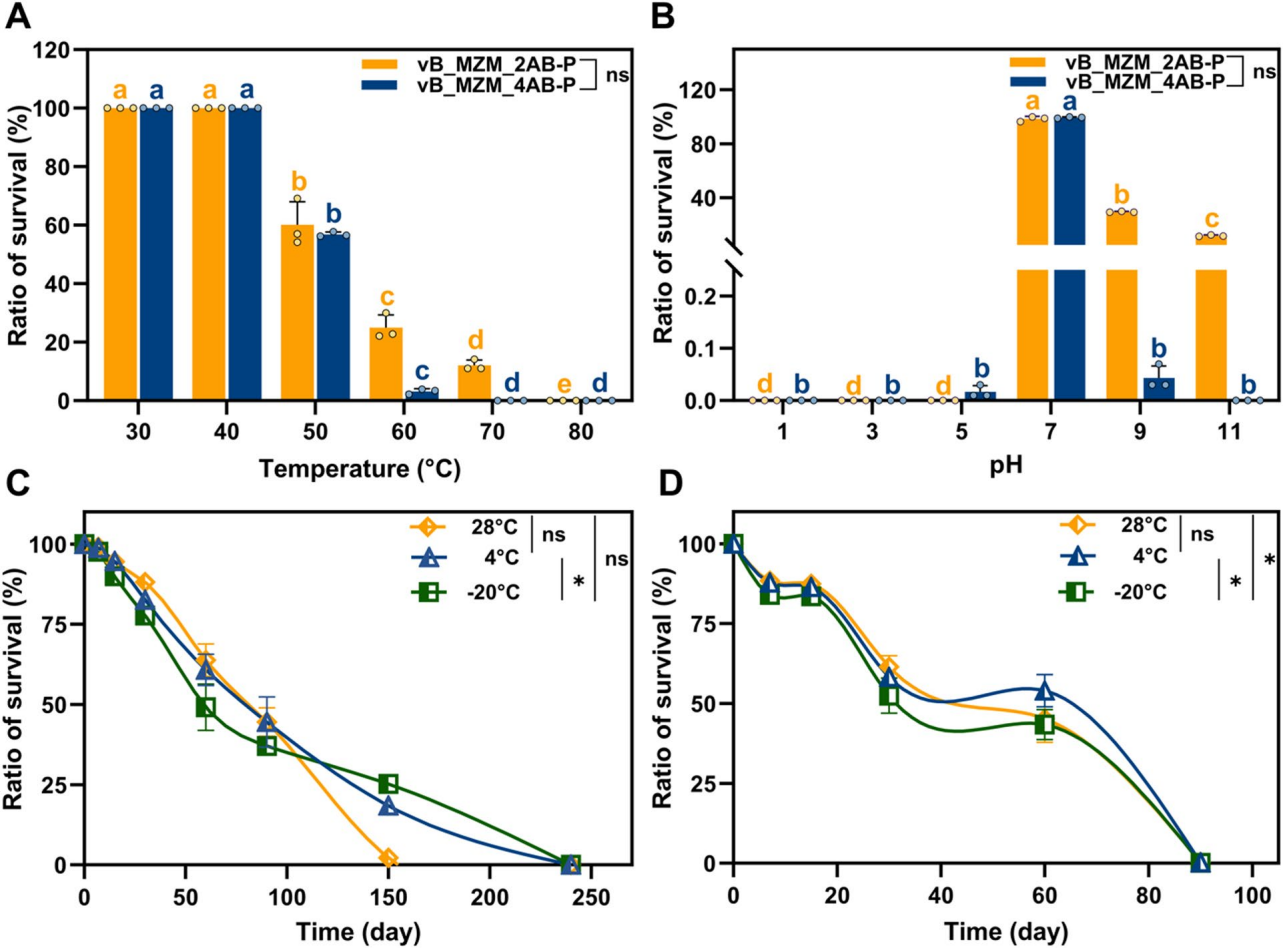
Stability assessment of phages vB_MZM_2AB-P and vB_MZM_4AB-P. (**A**) Thermal stability analysis, phage suspension was incubated at different temperatures for 30 min; (**B**) pH stability analysis, phage suspension was incubated at different pH values for 30 min; Storage stability analysis of phage vB_MZM_2AB-P (**C**) and vB_MZM_4AB-P (**D**). The results presented here are the mean values with SD indicated by error bars from three independent experiments. Different letters indicate significant differences (*p* < 0.05). The letters in orange and blue represent phage vB_MZM_2AB-P and vB_MZM_4AB-P, respectively. “ns” indicates no significant differences, *indicates significant differences (*p* < 0.05). The stability of both phages under different environmental conditions is evaluated, indicating their potential for practical applications.

To systematically evaluate the potential application value of the two phages, this study assessed their stability under various environmental conditions, including temperature, pH, and storage stability. The temperature stability results showed that phage vB_MZM_2AB-P had a survival rate of 24.90% at 60 °C, and maintained 12.40% of its activity at 70 °C (Fig. 3A). In contrast, phage vB_MZM_4AB-P exhibited a survival rate of 56.80% at 50 °C, but its activity decreased sharply at 60 °C, with only 3.20% survival (Fig. 3A). While vB_MZM_2AB-P showed a trend towards better thermal stability compared to vB_MZM_4AB-P under high-temperature conditions, however, no significant difference was observed overall (*p* = 0.15). Phage vB_MZM_2AB-P demonstrated good stability at neutral pH and retained a certain level of activity in alkaline environments. Even at pH 11.00, the phage maintained 11.90% activity (Fig. 3B). In contrast, phage vB_MZM_4AB-P exhibited stability only near neutral pH (pH = 7.00) and showed poor tolerance to pH changes (Fig. 3B). vB_MZM_2AB-P demonstrated a trend of superior pH tolerance compared to vB_MZM_4AB-P, but the difference was not statistically significant overall (*p* = 0.23). The storage stability tests showed that phage vB_MZM_2AB-P had a survival rate of 18.40% at 4 °C, and a survival rate of 25.20% after being stored at − 20 °C for 150 d. It remained active until 240 d of storage, making − 20 °C the most suitable storage condition for this phage (*p* < 0.05) (Fig. 3C). In contrast, phage vB_MZM_4AB-P exhibited survival rates of 43.40%, 54.00%, and 45.20% after 60 d of storage at − 20 °C, 4 °C, and 28 °C, respectively. However, its activity gradually diminished, and it lost all activity within three months. The optimal storage condition for vB_MZM_4AB-P is at 4 °C and 28 °C (*p* < 0.05) (Fig. 3D). These findings suggest that vB_MZM_2AB-P demonstrates relatively greater stability under long-term storage conditions than vB_MZM_4AB-P. In summary, both phages vB_MZM_2AB-P and vB_MZM_4AB-P demonstrate certain levels of thermal and storage stability. However, vB_MZM_2AB-P outperforms vB_MZM_4AB-P in terms of thermal stability, pH tolerance, and storage stability.

### Genomic characteristics of phages

To further investigate the genomic characteristics of phages vB_MZM_2AB-P and vB_MZM_4AB-P, this study analyzed their genomic structure and the functions of the encoded proteins (Table 1). The genome of vB_MZM_2AB-P contains a gene encoding tRNA-Arg, with the anticodon CCT, and three repeated sequences (Table S2). In vB_MZM_2AB-P, three repeat sequences were found at positions 13,563–13,589 (with a repeat unit of 8 bp and 100% match), 34,824–34,852 (with a repeat unit of 8 bp and 100% match), and 41,365–41,390 (with a repeat unit of 13 bp and 100% match) (Table S2). Similarly, the genome of vB_MZM_4AB-P also includes three repeated sequences (Table S3), but no tRNA genes were detected. In vB_MZM_4AB-P, three repeat regions were detected at positions 37,742–37,774 (with a repeat unit of 11 bp and 86% match), 37,742–37,811 (with a repeat unit of 23 bp and 76% match), and 38,201–38,228 (with a repeat unit of 11 bp and 100% match) (Table S3). These analyses highlight the presence of tandem repeat elements in the genomes of these bacteriophages, with some showing perfect conservation. The PhaBOX software predicted the lifestyle of the two phages as virulent (Table 1). Analysis using the PhageLeads software revealed no antibiotic resistance genes or virulence factors in the genomes of either vB_MZM_2AB-P or vB_MZM_4AB-P. These results suggest that the genomes of both phages have potential safety advantages, making them suitable for further biological applications.

**Table 1:** Genomic features of the isolated phages.

Features	Phage	
	vB_MZM_2AB-P	vB_MZM_4AB-P
Genome size (bp)	43,664	42,975
GC content (%)	47.76	40.57
Predicted genes	65	53
Hypothetical genes	49	22
Genes with predicted function	16	31
tRNA genes	1	None
Lifestyle	Virulent	Virulent
Antibiotic resistance genes	None	None
Virulence genes	None	None

**Fig. 4:**
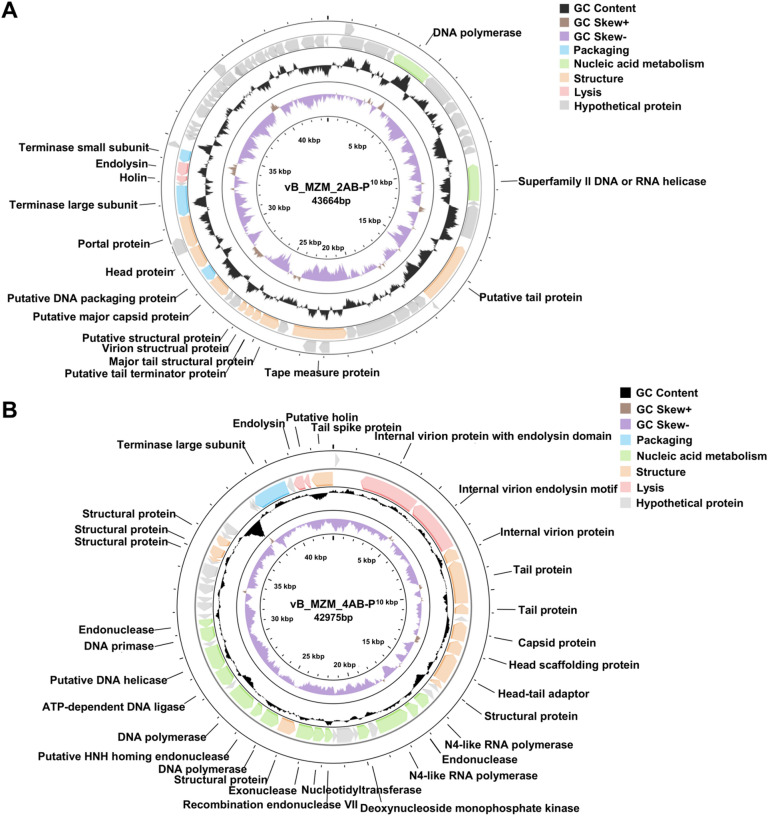
Genomic structural analysis of phages vB_MZM_2AB-P and vB_MZM_4AB-P. Schematic representation of the genome structure of phage vB_MZM_2AB-P (**A**) and vB_MZM_4AB-P (**B**). Different genetic modules are color-coded as follows: packaging gene module (blue), structural gene module (orange), cell lysis-related gene module (pink), and nucleic acid metabolism-related gene module (green). Genes of unknown function are represented in gray. GC skew is indicated in purple for leading strand gene modules and in brown for lagging strand gene modules. The detailed genomic organization of both phages is shown, with key functional regions and gene orientations highlighted.

Further visual analysis revealed that the proteins encoded by both phages exhibit a modular arrangement. The genome of phage vB_MZM_2AB-P is a linear double-stranded DNA, 43,664 bp in length (GenBank accession: PP091971), with a G + C content of 47.76% (Fig. 4A and Table 1). It encodes 65 open reading frames (ORFs), seven of which are transcribed in the forward direction, and 58 in the reverse direction (Table S4). Among these ORFs, 57 exhibit similarity to proteins from *A. baumannii* phages, while eight ORFs correspond to hypothetical proteins unique to vB_MZM_2AB-P. Functional annotation predicted that 16 ORFs encode proteins with known functions. Specifically, two ORFs (*gp*5 and *gp*12) encode nucleic acid metabolism-related proteins, including DNA polymerase and a superfamily II DNA or RNA helicase. Nine ORFs (*gp*15, *gp*22, *gp*26-29, *gp*32, *gp*34, and *gp*36) encode phage structural proteins, including tail proteins, tape measure protein, major tail structural protein, putative tail terminator protein, virion structural protein, structural protein, major capsid protein, head protein, and portal protein. Three ORFs (*gp*33, *gp*37, and *gp*41) encode phage packaging proteins, including DNA packaging protein, terminase large subunit, and terminase small subunit. Two ORFs (*gp*39 and *gp*40) encode core lysis-related proteins, including holin and endolysin.

The genome of phage vB_MZM_4AB-P is also a linear double-stranded DNA, 42,975 bp in length (GenBank accession: PP091972), with a G + C content of 40.57% (Fig. 4B and Table 1). This genome encodes 53 ORFs, one of which is transcribed in the forward direction and 52 in the reverse direction (Table S5). Of these ORFs, 46 exhibit similarity to proteins from *A. baumannii* phages; five ORFs correspond to hypothetical proteins unique to vB_MZM_4AB-P; one ORF shows similarity to proteins of *Pectobacterium* phage, and another ORF shares similarity with proteins from *Caryophanon* sp., which may originate from horizontal gene transfer. Thirty-one ORFs are predicted to encode proteins with known functions. Functional annotation results revealed that 12 ORFs (*gp*4-6, *gp*8-11, *gp*25, *gp*42-44, *gp*53) encode phage structural proteins, including internal virion protein, tail protein, capsid protein, head scaffolding protein, head–tail adaptor, structural protein, and tail spike protein, all transcribed in the same direction. Fourteen ORFs (*gp*14-16, *gp*18, *gp*22-24, *gp*26-28, *gp*30-31, *gp*33-34) encode nucleic acid metabolism-related proteins, such as N4-like RNA polymerase, endonuclease, deoxynucleoside monophosphate kinase, recombination endonuclease VII, nucleotidyltransferase, exonuclease, DNA polymerase, putative HNH homing endonuclease, ATP-dependent DNA ligase, putative DNA helicase, and DNA primase. One ORF (*gp*37) encodes a phage packaging protein, which includes the terminase large subunit. Four ORFs (*gp*2, *gp*3, *gp*51, and *gp*52) encode cell lysis-associated proteins, which include internal virion protein with an endolysin domain, internal virion endolysin motif, endolysin, and holin.

### Phylogenetic analysis of phages

To classify and identify phages vB_MZM_2AB-P and vB_MZM_4AB-P, this study employed a comprehensive approach combining BLASTn sequence similarity analysis from the NCBI database (Tables S6 and S7), in combination with the proteomic tree constructed by VipTree, a whole-genome phylogenetic tree based on Genome BLAST Distance Phylogeny (GBDP) method, the OrthoANI average nucleotide identity heatmap, the tANI (total average nucleotide identity) heatmap, and PCA scatter plot analysis.

**Fig. 5:**
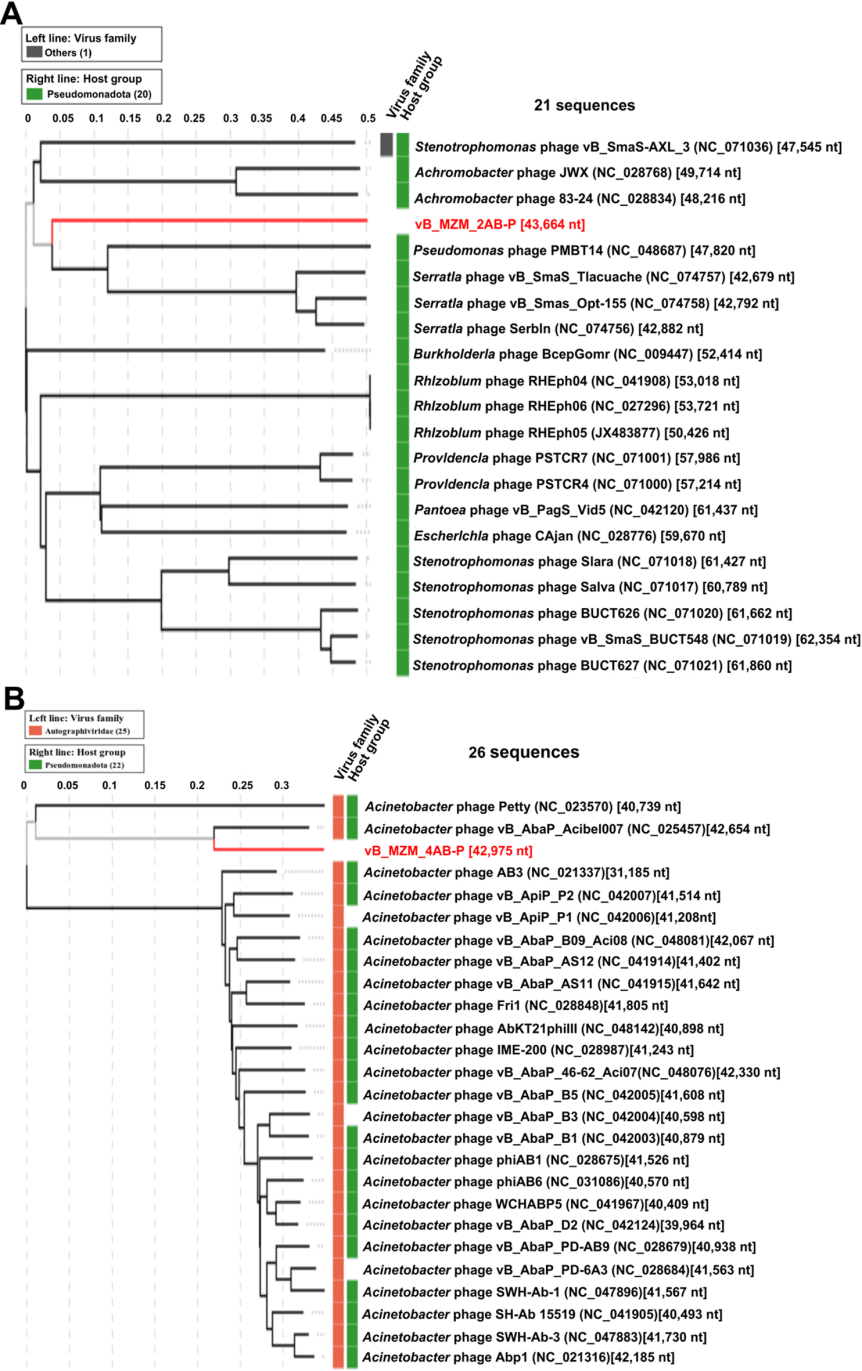
Proteomic tree of phages vB_MZM_2AB-P (**A**) and vB_MZM_4AB-P (**B**) constructed using VipTree. The tree includes all phages that exhibited a similarity score above zero. Phages marked with red squares belong to the Autographiviridae family, while phages marked with green squares have host bacteria classified under the Pseudomonadota phylum, and phages marked with gray squares belong to other families. The branch lengths shown in the figure represent the accumulated evolutionary distance along that branch, measured in the number of substitutions per site.

**Fig. 6:**
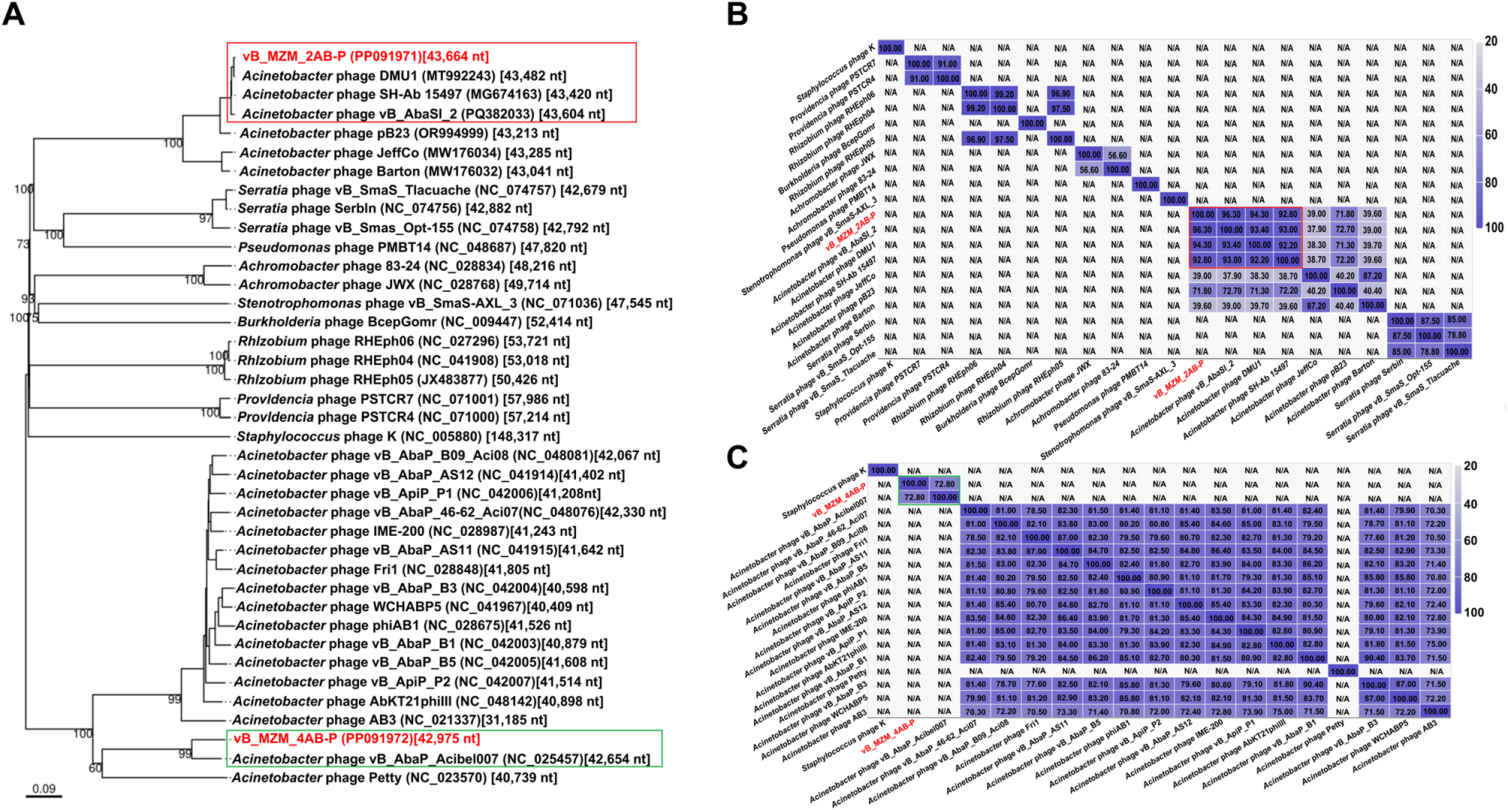
Phylogenetic and tANI analysis of phages related to vB_MZM_2AB-P and vB_MZM_4AB-P. A. Phylogenetic tree depicting the evolutionary relationships of phages with high BLASTN similarity to vB_MZM_2AB-P and vB_MZM_4AB-P. The tree was constructed using the VICTOR tool based on complete genomic sequences. Intergenomic distances were calculated and used to infer a balanced minimum evolution tree with branch support obtained from 100 pseudo-bootstrap replicates using FASTME (including SPR postprocessing for D0, D4, and D6 formulas)^23^. The tree is rooted at the midpoint and visualized with ggtree^24^. This tree illustrates the evolutionary placement of the novel phages, supporting their potential taxonomic distinctiveness. B, C. tANI analysis and clustering of phage genomes for vB_MZM_2AB-P (**B**) and vB_MZM_4AB-P (**C**) against related species. The analysis was performed using Vclust (https://afproject.org/vclust/), with clustering based on ICTV and MIUViG standards. Heatmaps visually represent the genomic similarity among the compared phages. These heatmaps confirm the genomic divergence of the novel phages, reinforcing their classification as new species and potential new genera.

Using the whole-genome tBLASTx method^18^, the proteomic phylogenetic tree generated by VipTree indicated that vB_MZM_2AB-P clustered within the same large branch as *Pseudomonas* phage PMBT14, *Serratia* phage vB_SmaS_Tlacuache, vB_SmaS_Opt-155, and Serbln (Fig. 5A). However, the heatmap analysis revealed that the OrthoANI values (OrthoANI values were generated by OAT software) of vB_MZM_2AB-P with these phages were all below 70.00% (Figure S2A and S2B), and the whole-genome similarity (BLASTn formula: Similarity = Query cover × Percent identity) was 0, suggesting significant genomic divergence. Further whole-genome BLASTn analysis identified phages with high similarity to vB_MZM_2AB-P, a whole-genome phylogenetic tree was constructed using the VICTOR web tool (Fig. 6A), along with a tANI heatmap. The whole-genome phylogenetic tree results show that vB_MZM_2AB-P is in the same major evolutionary branch as DMU1, SH-Ab15497, and vB_AbaSI_2, indicating a close phylogenetic relationship (Fig. 6A). The results showed that vB_MZM_2AB-P shared 98.10% (Similarity = Query Cover × Per. Ident, Query Cover = 99.00%, Per. Ident = 99.10%) similarity with vB_AbaSI_2, with corresponding tANI values of 96.30% (Fig. 6B). Phage vB_MZM_2AB-P shared 94.18% (Query Cover = 99.00%, Per. Ident = 95.13%) and 92.81% (Query Cover = 98.00%, Per. Ident = 94.70%) similarity with DMU1 and SH-Ab15497, respectively (Table S6), with corresponding tANI values of 94.30% and 92.80% (Fig. 6B). According to the phage classification criteria proposed by Dann Turner, similarity ≥ 95.00% defines the same species, ≥ 70.00% defines the same genus^19^, the similarity of vB_MZM_2AB-P with DMU1 and SH-Ab15497 was below 95.00%, and its genomic similarity with other *Acinetobacter* phages (pB23, Barton, and JeffCo) ranged from 18.53% to 68.01%. Moreover, there was no genomic similarity between vB_MZM_2AB-P and the *Pseudomonas* phage PMBT14, *Serratia* phage vB_SmaS_Tlacuache, vB_SmaS_Opt-155, or Serbln. Thus, from an evolutionary and taxonomic perspective, vB_MZM_2AB-P and vB_AbaSI_2 represents a novel species, which is consistent with the results from the PCA plot (Figure S3A). Notably, recent reports indicate that closely related phages, such as vB_AbaSI_2, DMU1, SH-Ab 15,497, and pB23, have not yet been clearly classified according to the latest taxonomic system^20–22^. Based on genomic similarity, phylogenetic positioning, and taxonomic threshold criteria, the available evidence currently supports designating vB_MZM_2AB-P as a member of a proposed novel genus within the Caudoviricetes order, Uroviricota class, Heunggongvirae realm of the Duplodnaviria domain. The proposed genus would be named *Acinetobactervirus*. Within this genus, both vB_AbaSI_2 and vB_MZM_2AB-P are proposed to belong to the same proposed species, provisionally named *Acinetobactervirus* vB_MZM_2AB-P.

The proteomic tree constructed using VipTree and the phylogenetic tree reveals that vB_MZM_4AB-P and *Acinetobacter* phage vB_AbaP_Acibel007 are positioned within the same evolutionary branch (Fig. 5B and 6A), with a whole-genome similarity of 73.82% (Query Cover = 83.00%, Per. Ident = 88.94%). The tANI and OrthoANI value between the two phages is 72.80% and 88.34% (Fig. 6C and S2C). According to the phage classification method established by Dann Turner et al*.*, the BLASTn genomic similarity between vB_MZM_4AB-P and *Acinetobacter* phage vB_AbaP_Acibel007 is less than 95.00%, categorizing vB_MZM_4AB-P as a novel species within the evolutionary classification, which is consistent with the results from the PCA plot (Figure S3B). Additionally, the similarity between vB_MZM_4AB-P and other phages, excluding *Acinetobacter* phage vB_AbaP_Acibel007, is consistently below 7.00%, indicating a high degree of genomic novelty. Based on the phylogenetic position and classification threshold criteria, vB_MZM_4AB-P is provisionally placed in the *Daemvirus* genus within the Beijerinckvirinae subfamily, Autographiviridae family, Caudoviricetes order, Uroviricota phylum, and Heunggongvirae realm of the Duplodnaviria domain. The proposed species name for *Daemvirus* vB_MZM_4AB-P is provisional.

**Fig. 7:**
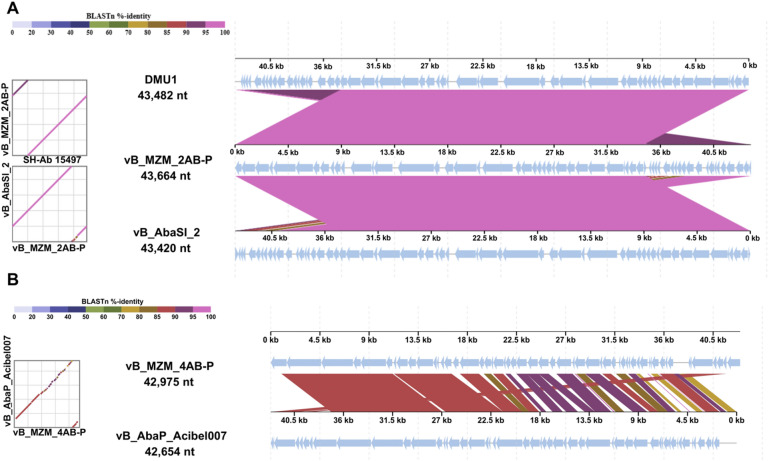
Collinearity analysis of phage genomes. (**A**) Genome collinearity analysis of phage vB_MZM_2AB-P with *Acinetobacter* phage SH-Ab15497 and DMU1. (**B**) Genome collinearity analysis of phage vB_MZM_4AB-P with *Acinetobacter* phage vB_AbaP_Acibel007. Results were generated using DiGAlign software. The color gradient from light purple to light reddish-purple indicates the gradual increase in BLASTn similarity from 0.00% to 100.00%. Reverse connecting lines represent genomic segment rearrangements. Arrow directions indicate the orientation of genomic sequences or genes, illustrating the relationship between different regions of the genome. The square diagram on the left corresponds to the arrows and lines in the collinearity plot on the right, showing the comparison and similarity between different genomes. The collinearity of phage genomes is displayed, showing sequence alignment and genomic rearrangements for comparative analysis.

A genome collinearity analysis was performed between phage vB_MZM_2AB-P and its closest phylogenetic relatives, *Acinetobacter* phages vB_AbaSI_2 and DMU1. The results revealed a high degree of genomic similarity across most regions (over 85.00% similarity, Fig. 7A), indicating a high level of evolutionary conservation among these phages. Notably, some regions of phage vB_AbaSI_2 exhibited up to 95.00% similarity with vB_MZM_2AB-P. Additionally, we identified a 9 kb genomic rearrangement in vB_MZM_2AB-P, primarily encoding proteins of unknown function. In contrast, the genome collinearity analysis between phage vB_MZM_4AB-P and Acibel007 demonstrated lower overall similarity, with several regions showing similarity below 80.00%, and multiple extended regions with no matching sequences (Fig. 7B). Specifically, several gene regions, including *gp*1, *gp*15, *gp*17, *gp*23, *gp*27, and *gp*53, lacked corresponding matches. These genes encode proteins of unknown function, Nucleotidyl transferase, putative HNH homing endonuclease, and tail spike protein. Compared to Acibel007, phage vB_MZM_4AB-P exhibited terminal gene rearrangements, with genes encoding terminase large subunit, endolysin, holin, tail spike protein, and proteins of unknown function.

To further clarify the evolutionary classification of vB_MZM_2AB-P and vB_MZM_4AB-P, this study constructed a phylogenetic tree based on the highly conserved characteristics of the DNA polymerase gene^25^, combined with the previously mentioned OrthoANI average nucleotide identity heatmap of phage genomes. The results show that the DNA polymerase Gp5 of phage vB_MZM_2AB-P is highly homologous with *Acinetobacter* phage SH-Ab15497 (99.36% similarity, Query Cover = 100.00%, Per. Ident = 99.36%) and DMU1 (99.20% similarity, Query Cover = 100.00%, Per. Ident = 99.20%), forming a closely related evolutionary branch (Figure S4). The DNA polymerase Gp26 of phage vB_MZM_4AB-P shares the highest similarity with *Acinetobacter* phage vB_AbaP_Acibel007 (96.82% similarity, Query Cover = 100.00%, Per. Ident = 96.82%), and they belong to the same evolutionary branch. This high similarity of conserved sequences not only corroborates the evolutionary conservation of phages vB_MZM_2AB-P and vB_MZM_4AB-P but also provides a reliable molecular clock calibration for their systematic classification. Furthermore, the phylogenetic tree based on DNA polymerase is in complete agreement with the whole-genome phylogenetic tree and the proteomic tree, supporting the reliability of the phage evolutionary relationships inferred in this study.

## Discussion

This study has successfully isolated and identified two lytic phages, vB_MZM_2AB-P and vB_MZM_4AB-P, targeting MDR-AB. ​Genomic and phylogenetic analyses reveal that these phages possess significant taxonomic novelty. Specifically, vB_MZM_2AB-P is tentatively classified as a new genus within Caudoviricetes, and vB_MZM_4AB-P is classified as a new species within the *Daemvirus* genus. ​This underscores the study’s contribution to expanding the known diversity of *Acinetobacter* phages. *A. baumannii*, as a globally recognized MDR pathogen, causes persistent infections that present significant challenges to public health, particularly in low- and middle-income countries^26^. Phage therapy, with its specific targeting and evolutionary adaptability, has emerged as a promising strategy to combat infections caused by drug-resistant bacteria^27,28^. In this study, both phages demonstrated potential antimicrobial activity and environmental stability, providing a basis for further exploration.

In terms of infection dynamics and environmental stability, vB_MZM_2AB-P and vB_MZM_4AB-P exhibit promising biological characteristics. Most *Acinetobacter* phages have latent periods of 10–30 min and lytic yields of 150–788 PFU/cell^22,29–38^ (Table S8). In this study, both phages exhibited relatively short latent period of 10 min, which is considered relatively short among known phages. For comparison, phages such as Abgy202141 and TCUAN2 showed extremely short latent period of 5 min, while others like vB_AbaS_qsb1, P425, vB_AbaAut_ChT04, P1068, Ab_WF01, and MRABP9 had latent periods of 10 min (Table S8). Phage vB_MZM_4AB-P demonstrated a high lytic capacity, reaching 746.70 PFU/cell, and the yield of vB_MZM_4AB-P is relatively higher than most phages reported in the literature such as vB_AbaS_SA1 (250 PFU/cell), P1068 (280 PFU/cell), Ab_WF01 (151 PFU/cell), pB23 (217 PFU/cell)vB_AbaAut_ChT04 (280 PFU/cell), vB_AbaM_ABMM1 (284 PFU/cell), MRABP9 (369 PFU/cell) and so on (Table S8). This efficient infection characteristic may be related to the lysis proteins encoded in their genomes^39^.

In terms of host range, vB_MZM_4AB-P exhibited a higher adsorption rate and within the panel of host strains tested in this study, showed lytic activity against a greater number of strains compared with vB_MZM_2AB‑P. Plaques formed by vB_MZM_4AB-P on *A. baumannii* 4AB plates have a central diameter of 1.25 mm and a halo diameter of 2.10 mm. Halos are frequently associated with exopolysaccharide depolymerase activity used by certain phages to expose cell-surface receptors^40^.

It was found in this study that the activity of phage vB_MZM_2AB-P and vB_MZM_4AB-P against *A.baumannii* is significantly reduced at pH values of 5 or lower (Fig. 3B). This highlights the importance of considering the pH of the infection site when evaluating the potential clinical application of phage therapy. *A. baumannii* is known to cause a variety of infections, including those in the lungs, blood, wounds, and central nervous system^41^. The pH values of alveolar cavities, blood, normal tissue fluids, and cerebrospinal fluid are typically close to neutral or slightly alkaline (pH 7.2–7.4), and rarely decrease significantly below pH 5, even during infection^41–43^. In contrast, the pH of urine can vary widely (5.0–8.0)^44^, and in certain urinary tract infections, an acidic environment may occur. However, *A. baumannii* has been isolated from urinary tract infections and catheter samples, where biofilm formation was shown to be optimal at 30 °C, pH 7.0, and a NaCl concentration of 5.0 g/L, reflecting a neutral pH in the urinary system^45^. Therefore, although the activity of the phage is weakened at low pH, its potential application in most common infection sites of *A. baumannii*, such as the lungs and blood, is unlikely to be significantly affected. Future studies are needed to evaluate the in vivo efficacy of these phages, particularly in environments that may present challenges such as low pH.​​

Regarding the MOI, the optimal MOI is highly phage-host specific, and different phages exhibiting a wide range of optimal MOIs, ranging from 0.001 to 10^46^. Both vB_MZM_2AB-P and vB_MZM_4AB-P exhibited good lytic activity at MOI = 1 and MOI = 10, comparable to other reported *A. baumannii* phages (e.g., Abgy202141, vB_AbaSt_W16, vB_AbaM_AB4P2) at MOI = 1 (Table S8). Based on the data from this study and the cited literature, an MOI of 1 appears to be an effective threshold for these particular phage-host pairs under the tested conditions, though this observation is context-dependent and not universally applicable. Additionally, vB_MZM_4AB-P showed comparable performance to other *A. baumannii* phages, such as TCUAN1 and vB_AbaM_ABMM1, at MOI = 10 (Table S8). In some application scenarios, such as in aquaculture environments, the MOI that yields the most significant control or therapeutic effects can also reach MOI = 10^47^. Therefore, MOI = 10 of phage vB_MZM_4AB-P (Fig. 2C) may be an empirical optimal value for this particular phage-host pair under the specific experimental conditions. It should be noted that at high MOIs, lysis from without may exert some impact on the bacterial killing effect. However, it is considered that this effect is not the primary mechanism under the experimental conditions investigated.

Phage cocktail can broaden lytic coverage across the tested strains. This study confirms that the combined use of vB_MZM_2AB-P and vB_MZM_4AB provided lytic activity against a larger subset of the tested strains than either phage alone. ​However, the small sample of six *A. baumannii* strains tested is a limitation and may not fully represent the diversity of this species. The narrow host range observed in this study highlights that therapeutic applications would require a much broader host range analysis. Further investigation with a broader range of *A. baumannii* strains is needed to better understand the full host specificity of these phages.

In terms of stability, vB_MZM_2AB-P exhibited significant tolerance to high temperatures and extreme pH (70 °C and pH = 11.00): it retained approximately 12.4% of its activity under 70 °C, higher than most similar phages (Table S8), demonstrating its potential application value in transport and high-temperature environments. In contrast, vB_MZM_4AB-P's thermal stability is more suited to conventional storage conditions (below 60 °C), and it exhibited more stable properties at neutral pH, making it suitable for regular refrigeration conditions. For long-term storage, vB_MZM_2AB-P can maintain activity for over 240 days at − 20 °C, comparable to other phages that can be stored long-term (e.g., vB_AbaM_AB4P2, vB_AbaP_ZC2) (Table S8). On the other hand, vB_MZM_4AB-P is better suited for short-term refrigeration, showing stability similar to that of phages typically stored under refrigeration.

vB_MZM_2AB-P employs a "holin-endolysin" dual lysis system (Gp39/Gp40), demonstrating the functional conservation of the classical dual system^48^. The holin protein encoded by the *gp*39 shares 100.00% sequence similarity with the holin protein of *Acinetobacter* phage DMU1 (GenBank accession: MT992243.1) (Query Cover = 100.00%, Per. Ident = 100.00%) (Figure S3). It contains a conserved domain (Figure S4A) that belongs to the Phage_holin_3_3 superfamily, and includes a transmembrane domain (Figure S4B), indicating its evolutionary stability in membrane pore formation. The endolysin encoded by the *gp*40 shows 99.52% similarity (Query Cover = 100.00%, Per. Ident = 99.52%) to the homologous protein of *Acinetobacter* phage DMU1 (GenBank accession: QOI69747.1) (Figure S3). This protein contains a zinc-dependent L-Ala-D-Glu peptidase domain, belonging to the Peptidase M15 superfamily C (Figure S5), which specifically hydrolyzes the peptide bonds containing L-alanine-D-glutamate in bacterial cell wall peptidoglycan. Although the holin and endolysin systems of vB_MZM_2AB-P and DMU1 are highly homologous, there are differences in their lytic spectra^20^. vB_MZM_2AB-P can lyse *A. baumannii* ATCC 19,606 and 2AB, but cannot lyse *A. baumannii* ATCC 17,978, while DMU1 can lyse both ATCC 17,978 and ATCC 19,606. This discrepancy may be due to differences in the lipid composition of the host bacterial membrane, which hindered the oligomerization of Gp39, preventing the transmembrane domain from effectively mediating membrane pore formation and impacting the lysis effect^49^. It could also be due to the host bacteria’s immune defense system developing specific resistance to phage infection. Another possibility is that the zinc finger domain of endolysin shows substrate affinity differentiation due to different chemical modifications of the host bacterial peptidoglycan, ultimately leading to the variation in the lytic spectrum. These differences in host range may also be attributed to differences in receptor-binding proteins^50^.

vB_MZM_4AB-P possesses a “multivariate regulation” system, exhibiting diverse lysis mechanisms. The phage genome contains genes *gp*2, *gp*3, *gp*51, and *gp*52, which participate in the cell lysis process. Among them, *gp*2 and *gp*3 encode internal virion proteins with an endolysin domain and an internal virion endolysin motif, respectively. The sequence similarities of *gp*2 and *gp*3 with the corresponding proteins of *Acinetobacter* phage vB_AbaP_Acibel007 (GenBank accession: NC_025457.1) are 96.66% (Query Cover = 100.00%, Percent Identity = 96.66%) and 96.58% (Query Cover = 100.00%, Percent Identity = 96.58%), respectively. The genes *gp*2 and *gp*3 may form a synergistic module, enhancing the lysis efficiency through a multi-protein complex. The gene *gp*51 encodes an endolysin, with the closest homology to the homologous protein of vB_AbaP_Acibel007 (Figure S3), showing a similarity of 88.14% (Query Cover = 100.00%, Per. Ident = 88.14%) (Figure S3). This protein is a type of glycoside hydrolase containing a conserved domain (Figure S6), belonging to the Glyco_hydro_19 Superfamily, and catalyzing the hydrolysis of the cross-linked region of the bacterial cell wall peptidoglycan, which may be related to the relatively broad-spectrum lytic ability of this phage. The gene *gp*52 encodes a holin, and the protein contains a transmembrane domain (Figure S7). It is highly similar to the holin protein of *Acinetobacter* phage vB_AbaP_Acibel007 (similarity 90.83%, Query Cover = 100.00%, Per. Ident = 90.83%), with the closest homology (Figure S3). The lysis-related proteins of vB_MZM_2AB-P and vB_MZM_4AB-P are highly conserved in their evolutionary relationships, which may be related to their key functions in the lysis process. This evolutionary conservation not only provides crucial clues for further research into phage lysis mechanisms but also offers new directions for the development of novel antimicrobial therapies.

When comparing the genome organizations of the two phages, differences in gene order were observed. However, this apparent gene fragment rearrangement may, at least in part, reflect variation in the chosen genome start positions during sequence assembly rather than true large-scale genomic rearrangements. Nonetheless, such differences in genome organization, whether due to true rearrangement events or reference coordinate variation, are commonly observed among phages and may still provide insights into their evolutionary dynamics^51^. In terms of taxonomy, the phylogenetic tree constructed based on the conserved protein DNA polymerase is highly consistent with the whole-genome phylogenetic tree. vB_MZM_2AB-P is tentatively classified as a new genus under the order Caudoviricetes. vB_MZM_4AB_P is classified as a new species within the *Daemvirus* genus under the Beijerinckvirinae subfamily of the Autographiviridae family, within the order Caudoviricetes, providing new perspectives for the taxonomic research of this order.

Phage therapy, owing to its high specificity and potential against antibiotic-resistant bacteria, is a promising alternative strategy. However, bacteria can evade phage infection through multiple mechanisms, including modification of surface receptors, such as lipopolysaccharides or capsular polysaccharides, to prevent phage adsorption^52^, inhibition of phage DNA injection^53^, activation of abortive infection mechanisms, the CRISPR/Cas system^54^, and toxin–antitoxin systems^55^. Phages counter bacterial resistance through evolutionary strategies such as modifying receptor-binding proteins (RBPs) to escape adsorption site recognition^56,57^, forming physical barriers to protect their genomes from nucleases^58^, secreting anti-CRISPR proteins or RNAs to inhibit CRISPR-Cas activity^59,60^, and suppressing bacterial DNA methylation systems to avoid recognition as foreign DNA^61^. Additionally, phage cocktails targeting multiple receptors or mechanisms can minimize resistance development under single-phage pressure, thereby enhancing therapeutic efficacy^52^. Therefore, the primary significance of this study lies in the isolation and genomic characterization of phages with novel genetic backgrounds, which contributes to expanding the phage library and provides new candidates for fundamental phage biology and ecology research.​​ Potential therapeutic applications would require extensive future investigation and validation.

## Conclusions

In this study, two lytic phages, vB_MZM_2AB-P and vB_MZM_4AB-P, targeting MDR-AB, were successfully isolated and characterized (Table S9). A comprehensive evaluation of their antimicrobial activity, environmental stability, and taxonomic features was conducted. The findings highlight their significance as novel biological entities with distinct properties. In terms of antibacterial performance, both phages exhibited short latent periods and efficient lytic activity. Regarding environmental stability, phage vB_MZM_2AB-P demonstrated robust stability under high temperature and strong alkaline conditions, making it a promising candidate for applications in the food industry, clinical disinfection, and medical device sterilization. On the other hand, phage vB_MZM_4AB-P displayed a broader lytic spectrum and higher lytic activity. These observations suggest that the distinct characteristics of the two phages may offer complementary advantages in certain contexts of practical use, although further studies are needed to substantiate their combined therapeutic potential. Furthermore, and most importantly, genomic and phylogenetic analyses confirmed the taxonomic novelty of these phages, with vB_MZM_2AB-P tentatively classified as likely representing a new genus and vB_MZM_4AB-P tentatively classified as a new species. This study expands the known diversity within the order Caudoviricetes and provides valuable genetic resources and insights for future phage biology and ecology research.

## Methods

### Isolation and purification of phage

Wastewater samples were collected from the drainage system beneath the overpass in Longhua District, Haikou City, Hainan Province, China. *A. baumannii* strains 2AB and 4AB were used as indicator bacteria. The sewage samples were centrifuged at 10,000 rpm for 15 min at 4 °C. Subsequently, 200 mL of the supernatant was transferred and mixed with 2 mL of indicator bacteria in 3 × LB medium. The mixture was incubated at 37 °C with shaking for 18–24 h, followed by further centrifugation. The supernatant was filtered through a 0.22 μm pore-size membrane filter to remove bacteria, and phages were then cultured using the double-layer agar plate method. For purification, a single, well-isolated plaque with a clear and regular morphology was selected for subsequent propagation. This single-plaque isolation procedure was repeated five times to ensure clonality. The purity of the phage isolate was confirmed by the consistent appearance of uniform plaques and further verified by transmission electron microscopy (TEM) to ensure a homogenous virion morphology.

### Morphological observation of phage

A fresh phage suspension was deposited onto a carbon-coated copper grid and negatively stained with 2.00% phosphotungstic acid^62^. The samples were sent to Zhongke Baice Technology Service Co., Ltd. (Beijing, China; https://www.zkbaice.cn/) for analysis. The morphology of the phage particles was observed using Transmission Electron Microscopy (TEM) at 100 kV (JEM-1400, JEOL, Japan). The dimensions of the phage head and tail were measured using ImageJ software.

### Determination of phage host range

To evaluate the lytic ability of the two phages against different bacterial strains, including *A. baumannii*, *Salmonella enterica*, *Klebsiella pneumoniae*, *Escherichia coli*, *Vibrio parahaemolyticus*, *Staphylococcus aureus*, *Pseudomonas aeruginosa*, *Bacillus subtilis*, and *Bacillus pumilus* (Table S1), a spot test was performed^63^. For this assay, 100 μL of bacterial suspension from each strain was mixed with semisolid LB agar and poured onto LB plates. After solidification, 10 μL of the gradient-diluted phage suspension was spotted onto the surface, with an equal volume of sterile water used as a control. The plates were incubated overnight, and the appearance of clear plaques was observed. The lytic effects were classified into three categories: an opaque zone, semi-confluent zone, and fully-lytic zone. Subsequently, the two phages were mixed in a 1:1 ratio, and the experiment was repeated to evaluate the lytic activity of the mixed phages.

### Determining the efficiency of plating (EOP)

To further evaluate the lytic effects of the two phages on the strains that tested positive in the spot test^64^, a defined volume of the diluted suspension was then mixed with both the host bacterial culture and the susceptible bacterial culture, followed by plating onto soft agar plates. After overnight incubation, plates with 30 to 300 plaques were counted, and the phage titer (PFU/mL) for each sample was determined. The Efficiency of Plating (EOP) was subsequently calculated using the formula:

$$\text{EOP}=\frac{\text{Titer of phage on test susceptible strain}}{\text{Titer of isolation host}}$$

The EOP ratio is interpreted as follows: ≥ 0.5 as high production efficiency, ≥ 0.1 to < 0.5 as medium production efficiency, 0.001 to 0.1 as low production efficiency, and ≤ 0.001 as inefficient.

### Antibiotic susceptibility testing of bacterial strains

This study employed the 2025 standards of the Clinical and Laboratory Standards Institute (CLSI) and used the Kirby-Bauer disc diffusion method to determine the inhibition zone diameters of *A. baumannii* against common antimicrobial agents for phage lysis spectrum analysis^65^. The antibiotics used include Piperacillin (PRL, 100 μg), Ampicillin-sulbactam (SAM, 10/10 μg), Piperacillin-tazobactam (TZP, 100/10 μg), Ceftazidime (CAZ, 30 μg), Cefepime (FEP, 30 μg), Cefotaxime (CTX, 30 μg), Ceftriaxone (CRO, 30 μg), Doripenem (DOR, 10 μg), Imipenem (IPM, 10 μg), Meropenem (MEM, 10 μg), Gentamicin (CN, 10 μg), Tobramycin (TOB, 10 μg), Amikacin (AK, 30 μg), Minocycline (MH, 30 μg), Ciprofloxacin (CIP, 5 μg), Levofloxacin (LEV, 5 μg), Gatifloxacin (GAT, 5 μg), Trimethoprim-Sulfamethoxazole (SXT, 1.25/23.75 μg), encompassing Penicillins, β-lactam combination agents, Cephems, Carbapenems, Aminoglycosides, Tetracyclines, Fluoroquinolones, and Folate pathway antagonists. In this study, MDR was defined as acquired non-susceptibility to at least one agent in three or more antimicrobial categories^66^. Resistance to imipenem and/or meropenem was defined as CRAB strains^67^.

### Analysis of optimal multiplicity of infection

According to previously reported methods^68^, exponentially growing *A. baumannii* strains 2AB and 4AB were separately mixed with phages at various optimal multiplicity of infection (MOIs) (10, 1, 0.1, 0.01, 0.001). The mixtures were incubated at 37 °C for 5 min to absorb, followed by centrifugation at 10,000 rpm for 1 min to remove unabsorbed phages. The pellets were then resuspended in fresh LB medium and incubated at 37 °C with shaking. The optical density at 600 nm (OD_*600*_) of the cultures was measured every 30 min, with bacterial cultures without added phage serving as the control. After 6 h of incubation, the mixture was centrifuged, filtered to remove bacteria, and the phage titer was quantified using the double-layer agar method.

### Determination of the phage adsorption rate

Phage suspensions and bacterial cultures were mixed in a 1:1 ratio and incubated for varying times. The mixture was then centrifuged at 10,000 rpm for 1 min. The phage titer in the supernatant was determined, and the phage adsorption rate was calculated using the following formula:

$$\text{Phage adsorption rate}=\frac{N}{{N}_{0}}\times 100\text{\%}$$

where *N*_*0*_ represents the phage titer at 0 min, while *N* represents the phage titer at time points of 1, 3, 5, 7, 10, 13, 15, 17, and 20 min^69^.

### One-step growth curve

Exponentially growing *A. baumannii* strains 2AB and 4AB were individually mixed with phages at an MOI of 1 and incubated for 5 min for adsorption. The mixture was then centrifuged at 10,000 rpm to remove unabsorbed phages. The pellets were resuspended in 50 mL of LB medium, and samples were taken at 5, 10, 15, 30–300 (at 30 min intervals) to measure changes in OD_*600*_ and phage titer. The experiment was performed three times. The results were used to generate the phage one-step growth curve. The average burst size was calculated as the final and the initial phage titers divided by the initial phage titer^70^. The phage titer is calculated as the mean number of plaques observed on plates at a given dilution, multiplied by the dilution factor, and divided by the inoculum volume (in mL), yielding the titer in PFU/mL. The one-step growth curve was generated as previously described^71^.

### Determination of the physical stability of the phage

Phage suspensions were incubated at various temperatures (30–80 °C) for 30 min, followed by cooling to room temperature. The phage titer was then measured, with room temperature serving as the control, and the experiment was repeated three times. For pH stability testing, phage suspensions were incubated for 30 min in buffers with pH values ranging from 1.00 to 11.00, with three replicates performed. The phage suspensions were stored at − 20 °C, 4 °C, and 28 °C, and titer changes were measured after 7, 14, 30, 60, 90, 150, and 240 d.

### Phage DNA extraction, sequencing, and genomic analysis

The genomic DNA of phage was extracted according to the Yuan et al*.* protocol^72^. The extracted DNA was subjected to whole-genome sequencing on the Illumina HiSeq platform at Sangon Biotechnology (Shanghai, China, https://www.sangon.com/). Libraries were constructed using the Hieff NGS® MaxUp II DNA Library Prep Kit for Illumina® reagent kit. Sequencing was performed with a 150 bp paired-end strategy, achieving a sequencing depth of approximately 100 × . Quality control and data trimming were performed using tools such as FastQC and Trimmomatic. Genome assembly was carried out using SPAdes 3.5.0. Coding sequences were predicted using FGENESV (Softberry, http://linux1.softberry.com), and visualization was done with CGView (https://proksee.ca/projects/new)^73,74^. The predicted genes were searched using the BLAST tool in the NCBI non-redundant protein sequences (NR) and Conserved Domain Database (CDD) to perform protein domain prediction (https://www.ncbi.nlm.nih.gov). Functional annotation was carried out using the Pfam database to identify protein sequences and their functional domains. tRNA genes were detected using tRNAscan-SE 2.0 (http://lowelab.ucsc.edu/tRNAscan-SE/index.html)^75^. Additionally, Tandem Repeat Finder was applied to identify repetitive sequences within the genome (https://tandem.bu.edu/trf/basic_submit)^76^. Potential virulence genes and resistance genes were analyzed using PhageLeads software (which utilizes the CARD, ResFinder, and VFDB databases) (https://phageleads.ku.dk/)^77^. Lifestyle of phage was predicted by PhaBOX v2 tool (https://phage.ee.cityu.edu.hk/phabox)^78^. The transmembrane domains were predicted using the Transmembrane Hidden Markov Model software (TMHMM 2.0) (https://services.healthtech.dtu.dk/service.php?TMHMM-2.0). Phylogenetic trees for conserved and functional genes were constructed using MEGA11 software, employing MUSCLE alignment and the neighbor-joining method with bootstrap analysis (1000 repetitions)^79^. Phylogenetic analysis of the phage genome was performed based on the tBLASTx algorithm using ViPTree (https://www.genome.jp/viptree/) to construct a proteomic tree and calculate genome sequence similarity^80^. The average nucleotide identity (ANI) tool was utilized to assess the genomic similarity between the phage genome and those of related species, followed by the construction of an OrthoANI heatmap to visualize the average nucleotide identity consistency using OAT software (https://www.ezbiocloud.net/tools/orthoani). OrthoANI, was developed to accommodate the concept of orthology for which both genome sequences were fragmented and only orthologous fragment pairs taken into consideration for calculating nucleotide identities^81^. A comparative phylogenetic analysis was conducted to explore the genetic relationships of the phages under study with related phages. The analysis was based on complete genomic sequences, and a phylogenetic tree was constructed using the VICTOR tool (https://ggdc.dsmz.de/victor.php) with the Genome BLAST distance phylogeny method. Genomes with the highest BLASTN similarity to the phages of interest were selected from the NCBI GenBank database via a BLASTN search. To assess the genomic similarity among the phages, total Average Nucleotide Identity (tANI) analysis was performed using the Vclust tool (https://afproject.org/vclust/), in accordance with ICTV and MIUViG standards. This analysis employed a k-mer size of 22, with a minimum k-mer count of 10, and a minimum sequence similarity threshold of 30%. The genomic clustering of the phages was carried out using Clusty, enabling the construction of a heatmap for visualizing genomic similarities. The resulting tANI values were then used to determine the degree of relatedness between the phages in this study and other related species. Genomic collinearity was analyzed using DiGAlign (https://www.genome.jp/digalign/)^82^.

### Statistical analysis

Statistical analyses were performed using SPSS (version 27.0) and GraphPad Prism (version 10.1.2). Differences between experimental treatments were assessed using one-way analysis of variance (ANOVA) followed by Duncan’s multiple range test for pairwise comparisons within groups (Fig. 2C, 3A, B). The mean differences between two independent groups were analyzed using an independent samples t-test (Fig. 3A, B). Two-way ANOVA was employed to assess the mean differences, considering the effects of two categorical independent variables on the dependent variable (Fig. 3C, D).

## Supplementary Information

Below is the link to the electronic supplementary material.

Supplementary Material 1

## Data Availability

All data generated or analyzed in this study are included in this article and its supplementary information files. The complete genome sequence of phage vB_MZM_2AB-P and vB_MZM_4AB-P is available at the NIH GenBank database (www.ncbi.nlm.nih.gov/genbank), under accession numbers PP091971 (vB_MZM_2AB-P) and PP091972 (vB_MZM_4AB-P).
