# Supplementary material for: Isolation and characterization of two novel phages with lytic activity against multidrug-resistant Acinetobacter baumannii strains: potential for phage therapy
Source: Sci Rep. 2025 Dec 10;15:43520. doi: 10.1038/s41598-025-27600-x (PMC12695905; doi:10.1038/s41598-025-27600-x)
Supplement: Supplementary file 1 — Supplementary Material 1 [file 41598_2025_27600_MOESM1_ESM.docx]

***Supplementary Materials***

**
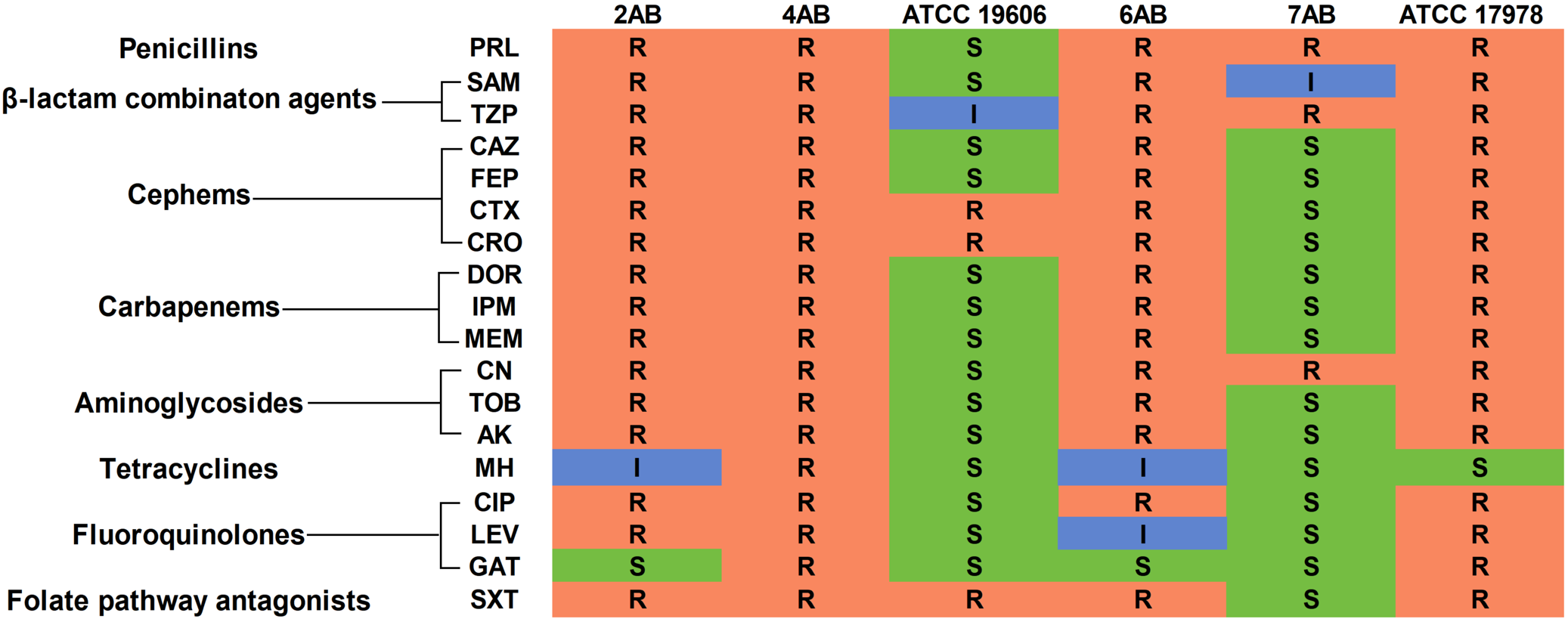
**

**Figure S1** Heatmap illustrating AST patterns. The antimicrobial susceptibility results are denoted by colored squares as follows: orange red for resistant (R), blue for intermediate (I), and green for susceptible (S).

**Table S1.** Host range analysis of phages vB_MZM_2AB-P and vB_MZM_4AB-P.

| **Strains** | **vB_MZM_2AB-P** | | **vB_MZM_4AB-P** | **vB_MZM_2AB-P+ vB_MZM_4AB-P** | **Strain resource** | **Host** |
| --- | --- | --- | --- | --- | --- | --- |
| *A. baumannii* 2AB ^a^ | +++ | ++ | | ++ | A clinical isolate ^1^ | Human |
| *A. baumannii* 4AB ^a^ | - | +++ | | +++ | A clinical isolate ^1^ | Human |
| *A. baumannii* ATCC-19606 ^a^ | ++ | - | | ++ | ATCC ^2^ |  |
| *A. baumannii* 6AB ^a^ | - | ++ | | ++ | A clinical isolate | Human |
| *A. baumannii* 7AB ^a^ | - | - | | - | A clinical isolate | Human |
| *A. baumannii* ATCC-17978 ^a^ | - | - | | - | ATCC ^3^ |  |
| *Salmonella enterica* CSV | - | - | | - | A laboratory isolate |  |
| *S. enterica* RSV | - | - | | - | A laboratory isolate |  |
| *S. enterica* NTV | - | - | | - | A laboratory isolate |  |
| *Klebsiella pneumoniae* Xu1 | - | - | | - | A laboratory isolate |  |
| *K. pneumoniae* ATCC-BAA-1705 | - | - | | - | ATCC ^4^ |  |
| *K. pneumoniae* ATCC-13883 | - | - | | - | ATCC ^5^ |  |
| *K. pneumoniae* 2106 ^a^ | - | - | | - | A clinical isolate ^6^ | Human |
| *K. pneumoniae* 0915 ^a^ | - | - | | - | A clinical isolate ^6^ | Human |
| *K. pneumoniae* 1025 ^a^ | - | - | | - | A clinical isolate ^6^ | Human |
| *Escherichia coli* CMCC 40396 | - | - | | - | CMCC ^7^ |  |
| *E. coli* 40102 | - | - | | - | A clinical isolate | Human |
| *E. coli* 40482 ^a^ | - | - | | - | A clinical isolate ^7^ | Human |
| *Vibrio parahaemolyticus* ATCC-17802 | - | - | | - | ATCC ^8^ |  |
| *Staphylococcus aureus* ATCC 6538 | - | - | | - | ATCC ^9^ |  |
| *S. aureus* TAO-1 | - | - | | - | A laboratory isolate |  |
| *Pseudomonas aeruginosa* XH | - | - | | - | A laboratory isolate |  |
| *Bacillus subtilis* CMCC 63501 | - | - | | - | CMCC ^10^ |  |
| *Bacillus pumilus* GR-8 | - | - | | - | ^11^ |  |

“a” indicates drug-resistant strains.

“−”, no clearing; “+”, opaque zone; “++”, semi-confluent zone; “+++”, fully-lytic zone.

**Table S2.** The repetitive sequence information of phage vB_MZM_2AB-P

| **Repetitive sequences (bp)** | **Period Size (bp)** | **Copy Number** | **Consensus Size (bp)** | **Percent Matches (%)** | **Percent Indels (%)** | **Score** | **A** | **C** | **G** | **T** | **Entropy (0-2)** |
| --- | --- | --- | --- | --- | --- | --- | --- | --- | --- | --- | --- |
| 13563-13589 | 8 | 3.4 | 8 | 100 | 0 | 54 | 0 | 29 | 33 | 37 | 1.58 |
| 34824-34852 | 8 | 3.6 | 8 | 100 | 0 | 58 | 37 | 37 | 24 | 0 | 1.56 |
| 41365-41390 | 13 | 2.0 | 13 | 100 | 0 | 52 | 15 | 23 | 23 | 38 | 1.92 |

**Table S3.** The repetitive sequence information of phage vB_MZM_4AB-P

| **Repetitive sequences (bp)** | **Period Size (bp)** | **Copy Number** | **Consensus Size (bp)** | **Percent Matches (%)** | **Percent Indels (%)** | **Score** | **A** | **C** | **G** | **T** | **Entropy (0-2)** |
| --- | --- | --- | --- | --- | --- | --- | --- | --- | --- | --- | --- |
| 37742-37774 | 11 | 2.8 | 12 | 86 | 9 | 50 | 36 | 21 | 0 | 42 | 1.53 |
| 37742-37811 | 23 | 3.0 | 22 | 76 | 15 | 70 | 34 | 21 | 2 | 41 | 1.68 |
| 38201-38228 | 11 | 2.5 | 11 | 100 | 0 | 56 | 42 | 39 | 0 | 17 | 1.50 |

**Table S4.** Protein functional annotation of phage vB_MZM_2AB-P

| **Gene accession** | **strand** | **Nucleotide position** | | **Length (amino acid）** | **Description** | **Best match** | **NCBI match accession** | **Similarity** | **E-value** |
| --- | --- | --- | --- | --- | --- | --- | --- | --- | --- |
|  |  | **start** | **end** |  |  |  |  |  |  |
| *gp*1 | - | 532 | 1689 | 385 | hypothetical protein | *Acinetobacter* phage DMU1 | QOI69782.1 | 93.51% | 0 |
| *gp*2 | + | 755 | 1165 | 136 | hypothetical protein | No hits |  | 0.00% |  |
| *gp*3 | - | 1702 | 2865 | 387 | hypothetical protein | *Acinetobacter* phage Barton | QXO06594.1 | 89.41% | 0 |
| *gp*4 | - | 2878 | 3180 | 100 | hypothetical protein | *Acinetobacter* phage Barton | QXO06593.1 | 68.37% | 2.00E-41 |
| *gp*5 | - | 3239 | 5113 | 624 | DNA polymerase | *Acinetobacter* phage DMU1 | QOI69779.1 | 99.20% | 0 |
| *gp*6 | - | 5117 | 6595 | 492 | hypothetical protein | *Acinetobacter* phage JeffCo | QXO06727.1 | 74.71% | 0 |
| *gp*7 | - | 6611 | 7135 | 174 | hypothetical protein | *Acinetobacter* phage SH-Ab 15497 | AUG85477.1 | 98.43% | 1.00E-126 |
| *gp*8 | - | 7195 | 7947 | 250 | hypothetical protein | *Acinetobacter* phage SH-Ab 15497 | AUG85476.1 | 98.00% | 0 |
| *gp*9 | - | 8064 | 8372 | 102 | hypothetical protein | *Acinetobacter* phage SH-Ab 15497 | AUG85475.1 | 97.06% | 1.00E-61 |
| *gp*10 | - | 8384 | 8920 | 178 | hypothetical protein | *Acinetobacter* phage DMU1 | QOI69774.1 | 95.51% | 2.00E-120 |
| *gp*11 | - | 8940 | 9278 | 112 | hypothetical protein | *Acinetobacter* phage SH-Ab 15497 | AUG85473.1 | 95.54% | 5.00E-73 |
| *gp*12 | - | 9796 | 11533 | 588 | superfamily II DNA or RNA helicase | *Acinetobacter* phage DMU1 | QOI69772.1 | 100.00% | 0 |
| *gp*13 | - | 11533 | 11730 | 65 | hypothetical protein | *Acinetobacter* phage DMU1 | QOI69771.1 | 100.00% | 1.00E-39 |
| *gp*14 | - | 11732 | 13321 | 529 | hypothetical protein | *Acinetobacter* phage SH-Ab 15497 | AUG85470.1 | 99.62% | 0 |
| *gp*15 | - | 13776 | 16670 | 964 | putative tail protein | *Acinetobacter* phage DMU1 | QOI69769.1 | 99.79% | 0 |
| *gp*16 | + | 16669 | 16800 | 43 | hypothetical protein | No hits |  | 0.00% |  |
| *gp*17 | - | 16820 | 17302 | 160 | hypothetical protein | *Acinetobacter* phage DMU1 | QOI69768.1 | 76.76% | 4.00E-88 |
| *gp*18 | - | 17232 | 17729 | 165 | hypothetical protein | *Acinetobacter* phage SH-Ab 15497 | AUG85467.1 | 99.39% | 2.00E-116 |
| *gp*19 | - | 17731 | 18471 | 246 | hypothetical protein | *Acinetobacter* phage SH-Ab 15497 | AUG85466.1 | 97.56% | 3.00E-179 |
| *gp20* | - | 18473 | 20449 | 658 | hypothetical protein | *Acinetobacter* phage SH-Ab 15497 | AUG85465.1 | 96.97% | 0 |
| *gp*21 | - | 20446 | 20916 | 156 | hypothetical protein | *Acinetobacter* phage DMU1 | QOI69764.1 | 99.36% | 1.00E-112 |
| *gp*22 | - | 20916 | 23498 | 860 | tape measure protein | *Acinetobacter* phage DMU1 | QOI69763.1 | 98.37% | 0 |
| *gp*23 | + | 21763 | 22230 | 155 | hypothetical protein | No hits |  | 0.00% |  |
| *gp*24 | + | 22354 | 22926 | 190 | hypothetical protein | No hits |  | 0.00% |  |
| *gp*25 | - | 23715 | 24185 | 156 | hypothetical protein | *Acinetobacter* phage SH-Ab 15497 | AUG85462.1 | 98.72% | 2.00E-106 |
| *gp*26 | - | 24188 | 25111 | 307 | major tail structural protein | *Acinetobacter* phage SH-Ab 15497 | AUG85461.1 | 99.02% | 0 |
| *gp*27 | - | 25114 | 25533 | 139 | putative tail terminator protein | *Acinetobacter* phage DMU1 | QOI69759.1 | 99.28% | 8.00E-97 |
| *gp*28 | - | 25520 | 25933 | 137 | virion structrual protein | *Acinetobacter* phage SH-Ab 15497 | AUG85459.1 | 100.00% | 9.00E-95 |
| *gp*29 | - | 25935 | 26288 | 117 | putative structural protein | *Acinetobacter* phage DMU1 | QOI69757.1 | 100.00% | 1.00E-78 |
| *gp*30 | - | 26285 | 26776 | 163 | hypothetical protein | *Acinetobacter* phage SH-Ab 15497 | AUG85457.1 | 100.00% | 7.00E-113 |
| *gp*31 | - | 26788 | 27060 | 90 | hypothetical protein | *Acinetobacter* phage SH-Ab 15497 | AUG85456.1 | 98.89% | 2.00E-57 |
| *gp*32 | - | 27096 | 28061 | 321 | putative major capsid protein | *Acinetobacter* phage DMU1 | QOI69754.1 | 100.00% | 0 |
| *gp*33 | - | 28061 | 28744 | 227 | putative DNA packaging protein | *Acinetobacter* phage DMU1 | QOI69753.1 | 100.00% | 1.00E-163 |
| *gp*34 | - | 28793 | 29857 | 354 | head protein | *Acinetobacter* phage SH-Ab 15497 | AUG85453.1 | 99.44% | 0 |
| *gp*35 | + | 29786 | 30517 | 243 | hypothetical protein | No hits |  | 0.00% |  |
| *gp*36 | - | 29857 | 31377 | 506 | portal protein | *Acinetobacter* phage SH-Ab 15497 | AUG85452.1 | 99.60% | 0 |
| *gp*37 | - | 31387 | 32847 | 486 | terminase large subunit | *Acinetobacter* phage SH-Ab 15497 | AUG85451.1 | 100.00% | 0 |
| *gp*38 | - | 32844 | 32984 | 46 | hypothetical protein | *Acinetobacter* phage DMU1 | QOI69749.1 | 97.00% | 4.00E-24 |
| *gp*39 | - | 32962 | 33309 | 115 | holin | *Acinetobacter* phage DMU1 | QOI69748.1 | 100.00% | 2.00E-78 |
| *gp*40 | - | 33313 | 33942 | 209 | endopeptidase | *Acinetobacter* phage DMU1 | QOI69747.1 | 99.52% | 1.00E-153 |
| *gp*41 | - | 33946 | 34545 | 199 | terminase small subunit | *Acinetobacter* phage DMU1 | QOI69746.1 | 98.99% | 1.00E-143 |
| *gp*42 | + | 34560 | 34811 | 83 | hypothetical protein | No hits |  | 0.00% |  |
| *gp*43 | - | 35026 | 35280 | 84 | hypothetical protein | *Acinetobacter* phage DMU1 | QOI69804.1 | 68.29% | 8.00E-34 |
| *gp*44 | - | 35211 | 35471 | 86 | hypothetical protein | *Acinetobacter* phage DMU1 | QOI69803.1 | 72.85% | 1.00E-39 |
| *gp*45 | - | 35452 | 35703 | 83 | hypothetical protein | *Acinetobacter* phage DMU1 | QOI69802.1 | 98.80% | 2.00E-54 |
| *gp*46 | - | 35703 | 35957 | 84 | hypothetical protein | *Acinetobacter* phage DMU1 | QOI69801.1 | 98.81% | 5.00E-56 |
| *gp*47 | - | 36211 | 36822 | 203 | hypothetical protein | *Acinetobacter* phage DMU1 | QOI69799.1 | 87.24% | 2.00E-128 |
| *gp*48 | - | 36822 | 37139 | 105 | hypothetical protein | *Acinetobacter* phage DMU1 | QOI69798.1 | 95.24% | 7.00E-69 |
| *gp*49 | - | 37142 | 37387 | 81 | hypothetical protein | *Acinetobacter* phage SH-Ab 15497 | AUG85496.1 | 95.06% | 6.00E-51 |
| *gp*50 | - | 37375 | 37773 | 132 | hypothetical protein | *Acinetobacter* phage DMU1 | QOI69796.1 | 85.61% | 2.00E-81 |
| *gp*51 | - | 37739 | 38209 | 156 | hypothetical protein | *Acinetobacter* phage DMU1 | QOI69795.1 | 66.25% | 9.00E-72 |
| *gp*52 | - | 38148 | 38813 | 221 | hypothetical protein | *Acinetobacter* phage DMU1 | QOI69794.1 | 96.83% | 1.00E-158 |
| *gp*53 | + | 38332 | 38793 | 153 | hypothetical protein | No hits |  | 0.00% |  |
| *gp*54 | - | 38907 | 39194 | 95 | hypothetical protein | *Acinetobacter* phage Barton | QXO06577.1 | 41.39% | 4.00E-15 |
| *gp*55 | - | 38953 | 39504 | 183 | hypothetical protein | *Acinetobacter* phage DMU1 | QOI69793.1 | 92.90% | 3.00E-124 |
| *gp*56 | - | 39491 | 39853 | 120 | hypothetical protein | *Acinetobacter* phage SH-Ab 15497 | AUG85491.1 | 98.33% | 1.00E-81 |
| *gp*57 | - | 39846 | 40064 | 72 | hypothetical protein | *Acinetobacter* phage DMU1 | QOI69791.1 | 91.67% | 3.00E-36 |
| *gp*58 | - | 40061 | 40549 | 162 | hypothetical protein | *Acinetobacter* phage DMU1 | QOI69790.1 | 98.77% | 2.00E-115 |
| *gp*59 | - | 40546 | 40743 | 65 | hypothetical protein | *Acinetobacter* phage DMU1 | QOI69789.1 | 92.31% | 2.00E-37 |
| *gp*60 | - | 40727 | 41152 | 141 | hypothetical protein | *Acinetobacter* phage SH-Ab 15497 | AUG85487.1 | 97.87% | 2.00E-98 |
| *gp*61 | - | 41085 | 41525 | 146 | hypothetical protein | *Acinetobacter* phage DMU1 | QOI69787.1 | 97.26% | 3.00E-103 |
| *gp*62 | - | 41570 | 42259 | 229 | hypothetical protein | *Acinetobacter* phage SH-Ab 15497 | AUG85486.1 | 97.38% | 4.00E-163 |
| *gp*63 | - | 42378 | 43004 | 208 | hypothetical protein | *Acinetobacter* phage SH-Ab 15497 | AUG85485.1 | 88.46% | 6.00E-136 |
| *gp*64 | - | 43006 | 43473 | 155 | hypothetical protein | *Acinetobacter* phage SH-Ab 15497 | AUG85484.1 | 100.00% | 8.00E-113 |
| *gp*65 | - | 43529 | 43660 | 43 | hypothetical protein | No hits |  | 0.00% | 0 |

**Table S5.** Protein functional annotation of phage vB_MZM_4AB-P

| **Gene accession** | **strand** | **Nucleotide position** | | **Length (amino acid）** | **Description** | **Best match** | **NCBI match accession** | **Similarity** | **E-value** |
| --- | --- | --- | --- | --- | --- | --- | --- | --- | --- |
|  |  | **start** | **end** |  |  |  |  |  |  |
| *gp*1 | + | 120 | 278 | 130 | hypothetical protein | No hits |  | 98.21% | 3.00E-162 |
| *gp*2 | - | 1456 | 4605 | 3745 | internal virion protein with endolysin domain | *Acinetobacter* phage vB_AbaP_Acibel007 | YP_009103256.1 | 96.66% | 0 |
| *gp*3 | - | 4625 | 7522 | 3406 | internal virion lysozyme motif | *Acinetobacter* phage vB_AbaP_Acibel007 | YP_009103255.1 | 96.58% | 0 |
| *gp*4 | - | 7538 | 8239 | 821 | internal virion protein | *Acinetobacter* phage vB_AbaP_Acibel007 | YP_009103254.1 | 91.88% | 1.00E-132 |
| *gp*5 | - | 8241 | 10550 | 1975 | tail protein | *Acinetobacter* phage vB_AbaP_Acibel007 | YP_009103253.1 | 93.37% | 0 |
| *gp*6 | - | 10561 | 11118 | 558 | tail protein | *Acinetobacter* phage vB_AbaP_Acibel007 | YP_009103252.1 | 98.38% | 2.00E-132 |
| *gp*7 | - | 11245 | 11475 | 507 | hypothetical protein | *Acinetobacter* phage vB_AbaP_Acibel007 | YP_009103250.1 | 81.58% | 8.00E-28 |
| *gp*8 | - | 11523 | 12533 | 1405 | capsid protein | *Acinetobacter* phage vB_AbaP_Acibel007 | YP_009103249.1 | 93.75% | 0 |
| *gp*9 | - | 12552 | 13292 | 1149 | head scaffolding protein | *Acinetobacter* phage vB_AbaP_Acibel007 | YP_009103248.1 | 95.12% | 2.00E-167 |
| *gp*10 | - | 13305 | 14855 | 1455 | head-tail adaptor | *Acinetobacter* phage vB_AbaP_Acibel007 | YP_009103247.1 | 98.26% | 0 |
| *gp*11 | - | 14865 | 15188 | 424 | structural protein | *Acinetobacter* phage vB_AbaP_Acibel007 | YP_009103246.1 | 93.46% | 6.00E-64 |
| *gp*12 | - | 15198 | 15371 | 192 | hypothetical protein | *Acinetobacter* phage vB_AbaP_Acibel007 | YP_009103245.1 | 96.49% | 8.00E-33 |
| *gp*13 | - | 15548 | 15853 | 389 | hypothetical protein | *Acinetobacter* phage vB_AbaP_Acibel007 | YP_009103244.1 | 82.18% | 4.00E-51 |
| *gp*14 | - | 15871 | 16542 | 827 | N4-like RNA polymerase | *Acinetobacter* phage vB_AbaP_Acibel007 | YP_009103243.1 | 91.93% | 1.00E-147 |
| *gp*15 | - | 16675 | 17196 | 522 | endonuclease | *Pectobacterium* phage PPWS1 | YP _009785664.1 | 52.91% | 4.00E-52 |
| *gp*16 | - | 17295 | 19061 | 1607 | N4-like RNA polymerase | *Acinetobacter* phage vB_AbaP_Acibel007 | YP_009103243.1 | 93.76% | 0 |
| *gp*17 | - | 19061 | 19513 | 366 | hypothetical protein | *Acinetobacter* phage vB_AbaP_WU2001 | QVQ34714.1 | 46.50% | 1.00E-41 |
| *gp*18 | - | 19518 | 20162 | 810 | deoxynucleoside monophosphate kinase | *Acinetobacter* phage AJO2 | AYJ76513.1 | 94.39% | 6.00E-146 |
| *gp*19 | - | 20159 | 20353 | 195 | hypothetical protein | Caryophanon sp. | MBS7344303.1 | 41.18% | 7.00E-08 |
| *gp20* | - | 20356 | 21300 | 945 | hypothetical protein | *Acinetobacter* phage vB_AbaP_Acibel007 | YP_009103241.1 | 94.86% | 0 |
| *gp*21 | - | 21281 | 21499 | 234 | hypothetical protein | No hits |  | 0.00% |  |
| *gp*22 | - | 21483 | 21932 | 584 | recombination endonuclease VII | *Acinetobacter* phage AJO2 | AYJ76511.1 | 98.66% | 3.00E-105 |
| *gp*23 | - | 21925 | 22533 | 644 | Nucleotidyl transferase | *Acinetobacter* phage vB_AbaP_Acibel007 | YP_009103238.1 | 37.66% | 1.00E-30 |
| *gp*24 | - | 22463 | 23431 | 1075 | exonuclease | *Acinetobacter* phage vB_AbaP_Acibel007 | YP_009103237.1 | 93.75% | 0 |
| *gp*25 | - | 23514 | 24440 | 1153 | structural protein | *Acinetobacter* phage vB_AbaP_Acibel007 | YP_009103235.1 | 96.75% | 0 |
| *gp*26 | - | 24487 | 25431 | 891 | DNA polymerase | *Acinetobacter* phage vB_AbaP_Acibel007 | YP_009103233.1 | 96.82% | 0 |
| *gp*27 | - | 25517 | 26044 | 458 | putative HNH homing endonuclease | *Acinetobacter* phage APK20 | UAW10060.1 | 88.57% | 6.00E-116 |
| *gp*28 | - | 26138 | 27499 | 1570 | DNA polymerase | *Acinetobacter* phage AJO2 | AYJ76516.1 | 96.47% | 0 |
| *gp*29 | - | 27608 | 27811 | 269 | hypothetical protein | No hits |  | 0.00% |  |
| *gp*30 | - | 27804 | 28787 | 972 | ATP-dependent DNA ligase | *Acinetobacter* phage vB_AbaP_Acibel007 | YP_009103231.1 | 77.95% | 0 |
| *gp*31 | - | 28771 | 30105 | 1437 | putative DNA helicase | *Acinetobacter* phage vB_AbaP_Acibel007 | YP_009103229.1 | 98.20% | 0 |
| *gp*32 | - | 30107 | 30340 | 276 | hypothetical protein | No hits |  | 0.00% |  |
| *gp*33 | - | 30342 | 31145 | 398 | DNA primase | *Acinetobacter* phage vB_AbaP_Acibel007 | YP_009103227.1 | 92.30% | 0 |
| *gp*34 | - | 31132 | 31581 | 335 | endonuclease | *Acinetobacter* phage vB_AbaP_B1 | YP_009610314.1 | 42.67% | 7.00E-29 |
| *gp*35 | - | 31591 | 31854 | 248 | hypothetical protein | *Acinetobacter* phage vB_AbaP_Acibel007 | YP_009103226.1 | 87.36% | 8.00E-47 |
| *gp*36 | - | 32011 | 32475 | 617 | hypothetical protein | *Acinetobacter* phage vB_AbaP_Acibel007 | YP_009103224.1 | 80.19% | 5.00E-87 |
| *gp*37 | - | 32465 | 32761 | 393 | hypothetical protein | No hits |  | 0.00% |  |
| *gp*38 | - | 32905 | 33444 | 787 | hypothetical protein | *Acinetobacter* phage vB_AbaP_Acibel007 | YP_009103222.1 | 87.71% | 3.00E-111 |
| *gp*39 | - | 33454 | 33765 | 358 | hypothetical protein | *Acinetobacter* phage vB_AbaP_Acibel007 | YP_009103220.1 | 73.79% | 7.00E-50 |
| *gp*40 | - | 33749 | 34495 | 881 | hypothetical protein | *Acinetobacter* phage vB_AbaP_Acibel007 | YP_009103219.1 | 40.06% | 4.00E-49 |
| *gp*41 | - | 34602 | 34721 | 168 | hypothetical protein | *Acinetobacter* phage vB_AbaP_Acibel007 | YP_009103218.1 | 71.79% | 9.00E-11 |
| *gp*42 | - | 34721 | 34870 | 134 | structural protein | *Acinetobacter* phage vB_AbaP_Acibel007 | YP_009103216.1 | 70.37% | 1.00E-14 |
| *gp*43 | - | 34863 | 35504 | 688 | structural protein | *Acinetobacter* phage vB_AbaP_Acibel007 | YP_009103215.1 | 59.37% | 2.00E-83 |
| *gp*44 | - | 35589 | 36089 | 591 | structural protein | *Acinetobacter* phage vB_AbaP_Acibel007 | YP_009103214.1 | 86.71% | 4.00E-101 |
| *gp*45 | - | 36097 | 36306 | 193 | hypothetical protein | *Acinetobacter* phage vB_AbaP_Acibel007 | YP_009103213.1 | 76.81% | 2.00E-31 |
| *gp*46 | - | 36323 | 36841 | 818 | hypothetical protein | *Acinetobacter* phage vB_AbaP_Acibel007 | YP_009103212.1 | 92.44% | 1.00E-112 |
| *gp*47 | - | 38230 | 38427 | 296 | hypothetical protein | *Acinetobacter* phage vB_AbaP_Acibel007 | YP_009103264.1 | 87.69% | 2.00E-28 |
| *gp*48 | - | 38414 | 38533 | 209 | hypothetical protein | *Acinetobacter* phage vB_AbaP_Acibel007 | YP_009103263.1 | 71.47% | 2.00E-08 |
| *gp*49 | - | 38530 | 40458 | 1878 | terminase large subunit | *Acinetobacter* phage vB_AbaP_Acibel007 | YP_009103262.1 | 96.11% | 0 |
| *gp*50 | - | 40468 | 40824 | 450 | hypothetical protein | *Acinetobacter* phage AJO2 | AYJ76507.1 | 99.15% | 5.00E-78 |
| *gp*51 | - | 40827 | 41411 | 756 | lysozyme | *Acinetobacter* phage AJO2 | AYJ76508.1 | 97.42% | 1.00E-136 |
| *gp*52 | - | 41398 | 41724 | 449 | putative holin | *Acinetobacter* phage vB_AbaP_Acibel007 | YP_009103258.1 | 90.83% | 3.00E-63 |
| *gp*53 | - | 41800 | 42954 | 782 | tail spike protein | *Acinetobacter* phage APK37.1 | UAW07728.1 | 89.84% | 0 |

**Table S6.** Summary of comparative genomic analysis of phage vB_MZM_2AB-P

| **Phage** | **NCBI accession** | **Sequence length (bp)** | **GC (%)** | **Similarity (%)** ^a^ | **Reference** |
| --- | --- | --- | --- | --- | --- |
| *Acinetobacter* phage vB_AbaSl_2 | PQ382033.1 | 43,604 | 48.00 | 98.11 |  |
| *Acinetobacter* phage DMU1 | MT992243.1 | 43,482 | 47.83 | 94.18 | ^12^ |
| *Acinetobacter* phage SH-Ab 15497 | MG674163.1 | 43,420 | 47.87 | 92.81 | ^13^ |
| *Acinetobacter* phage pB23 | OR994999.1 | 43,213 | 47.10 | 68.01 |  |
| *Acinetobacter* phage Barton | MW176032.1 | 43,041 | 47.72 | 29.49 |  |
| *Acinetobacter* phage JeffCo | MW176034.1 | 43,285 | 47.80 | 18.53 |  |
| *Pseudomonas* phage PMBT14 | NC_048687 | 47,820 | 55.00 | 0.00 |  |
| *Serratia* phage vB_SmaS_Tlacuache | NC_074757.1 | 42,679 | 52.00 | 0.00 |  |
| *Serratia* phage vB_SmaS_Opt-155 | NC_074758 | 42,792 | 52.00 | 0.00 |  |
| *Serratia* phage Serbln | NC_074756.1 | 42,882 | 52.00 | 0.00 | ^14^ |
| *Pseudomonas* phage PMBT14 | NC_048687 | 47,820 | 55.00 | 0.00 |  |
| *Achromobacter* phage 83-24 | NC_028834 | 48,216 | 55.00 | 0.00 | ^15^ |
| *Achromobacter* phage JWX | NC_028768 | 49,714 | 55.00 | 0.00 | ^15^ |
| *Stenotrophomonas* phage vB_SmaS-AXL_3 | NC_071036 | 47,545 | 63.00 | 0.00 | ^16^ |
| *Burkholderla* phage BcepGomr | NC_009447 | 52,414 | 56.00 | 0.00 | ^15^ |
| *Rhlzobium* phage RHEph06 | NC_027296 | 53,721 | 56.00 | 0.00 |  |
| *Rhlzobium* phage RHEph04 | NC_041908 | 53,018 | 56.00 | 0.00 |  |
| *Rhlzobium* phage RHEph05 | JX483877 | 50,426 | 56.00 | 0.00 |  |
| *Provldencia* phage PSTCR7 | NC_071001 | 57,986 | 37.00 | 0.00 |  |
| *Provldencla* phage PSTCR4 | NC_071000 | 57,214 | 37.00 | 0.00 |  |
| *Staphylococcus* phage K | NC_005880 | 148,317 | 30.00 | 0.00 |  |

^a^ represents genomic similarity, calculated as BLASTN Similarity = Query cover × Percent identity.

**Table S7.** Summary of comparative genomic analysis of phage vB_MZM_4AB-P

| **Phage** | **NCBI Accession** | **Sequence Length (bp)** | **GC (%)** | **Similarity (%)^a^** | **Reference** |
| --- | --- | --- | --- | --- | --- |
| *Acinetobacter* phage Petty | NC_023570 | 40,739 | 42.19 | 3.06 |  |
| *Acinetobacter* phage vB_AbaP_Acibel007 | NC_025457 | 42,654 | 41.17 | 72.93 | ^17^ |
| *Acinetobacter* phage AB3 | NC_021337 | 31,185 | 39.18 | 0.73 |  |
| *Acinetobacter* phage vB_ApiP_P2 | NC_042007 | 41,514 | 39.33 | 0.73 |  |
| *Acinetobacter* phage vB_ApiP_P1 | NC_042006 | 41,208 | 39.20 | 0.00 |  |
| *Acinetobacter* phage vB_AbaP_B09_Aci08 | NC_048081 | 42,067 | 39.28 | 3.67 |  |
| *Acinetobacter* phage vB_AbaP_AS12 | NC_041914 | 41,402 | 39.31 | 2.21 | ^18^ |
| *Acinetobacter* phage vB_AbaP_AS11 | NC_041915 | 41,642 | 39.29 | 0.74 | ^18^ |
| *Acinetobacter* phage Fri1 | NC_028848 | 41,805 | 39.29 | 0.73 |  |
| *Acinetobacter* phage AbKT21phiIII | NC_048142 | 40,898 | 39.36 | 0.73 | ^19^ |
| *Acinetobacter* phage IME-200 | NC_028987 | 41,243 | 39.31 | 0.72 |  |
| *Acinetobacter* phage vB_AbaP_46-62_Aci07 | NC_048076 | 42,330 | 39.14 | 0.74 |  |
| *Acinetobacter* phage vB_AbaP_B5 | NC_042005 | 41,608 | 39.31 | 0.73 |  |
| *Acinetobacter* phage phiAB1 | NC_028675 | 41,526 | 39.09 | 2.22 | ^20^ |
| *Acinetobacter* phage vB_AbaP_B1 | NC_042003 | 40,879 | 39.14 | 2.21 |  |
| *Acinetobacter* phage vB_AbaP_B3 | NC_042004 | 40,598 | 39.28 | 2.21 |  |
| *Acinetobacter* phage phiAB6 | NC_031086 | 40,570 | 39.47 | 0.73 |  |
| *Acinetobacter* phage WCHABP5 | NC_041967 | 40,409 | 39.39 | 0.74 |  |
| *Acinetobacter* phage vB_AbaP_D2 | NC_042124 | 39,964 | 39.23 | 1.45 | ^21^ |
| *Acinetobacter* phage vB_AbaP_PD-AB9 | NC_028679 | 40,938 | 39.34 | 0.74 |  |
| *Acinetobacter* phage vB_AbaP_PD-6A3 | NC_028684 | 41,563 | 39.48 | 0.74 | ^22^ |
| *Acinetobacter* phage SWH-Ab-1 | NC_047896 | 41,567 | 39.42 | 0.73 |  |
| *Acinetobacter* phage SH-Ab 15519 | NC_041905 | 40,493 | 39.46 | 0.73 |  |
| *Acinetobacter* phage SWH-Ab-3 | NC_047883 | 41,730 | 39.38 | 0.00 |  |
| *Acinetobacter* phage Abp1 | NC_021316 | 42,185 | 39.15 | 0.00 |  |

**^a^** represents genomic similarity, calculated as BLASTN Similarity = Query cover × Percent identity.

**
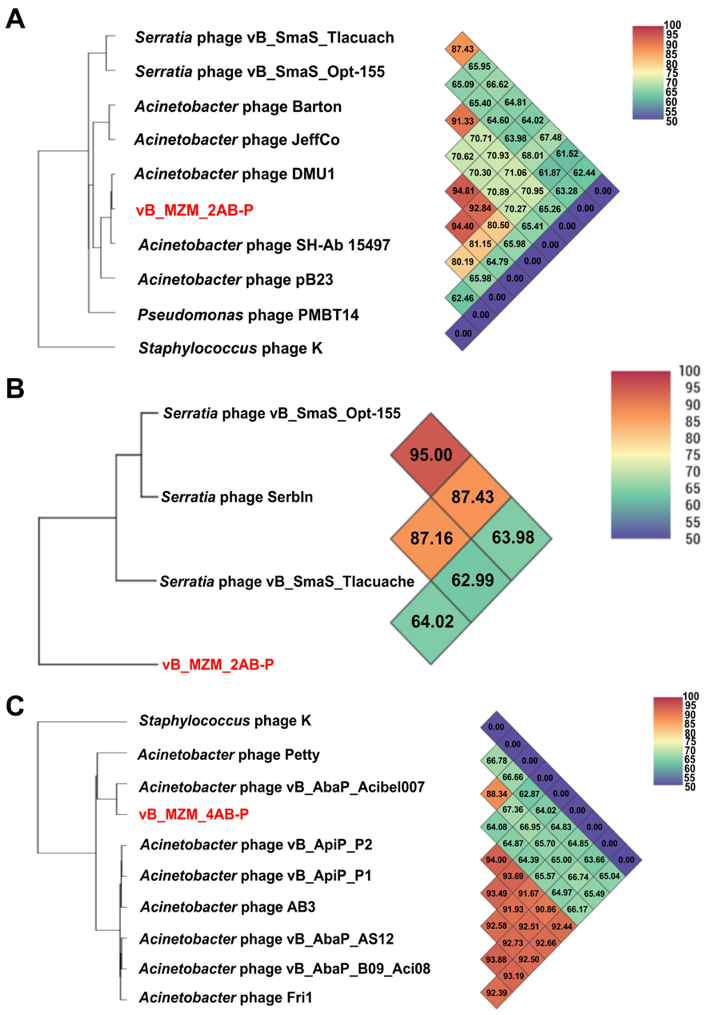
**

**Figure S2.** Genomic similarity heatmap of phage vB_MZM_2AB-P (A, B) and vB_MZM_4AB-P (C) generated based on OrthoANI values calculated using the OAT software. The color gradient from purple to red represents an increase in OrthoANI values from 50% to 100%. Phages analyzed in this study are highlighted in red. (OrthoANI values were generated by OAT software).

**
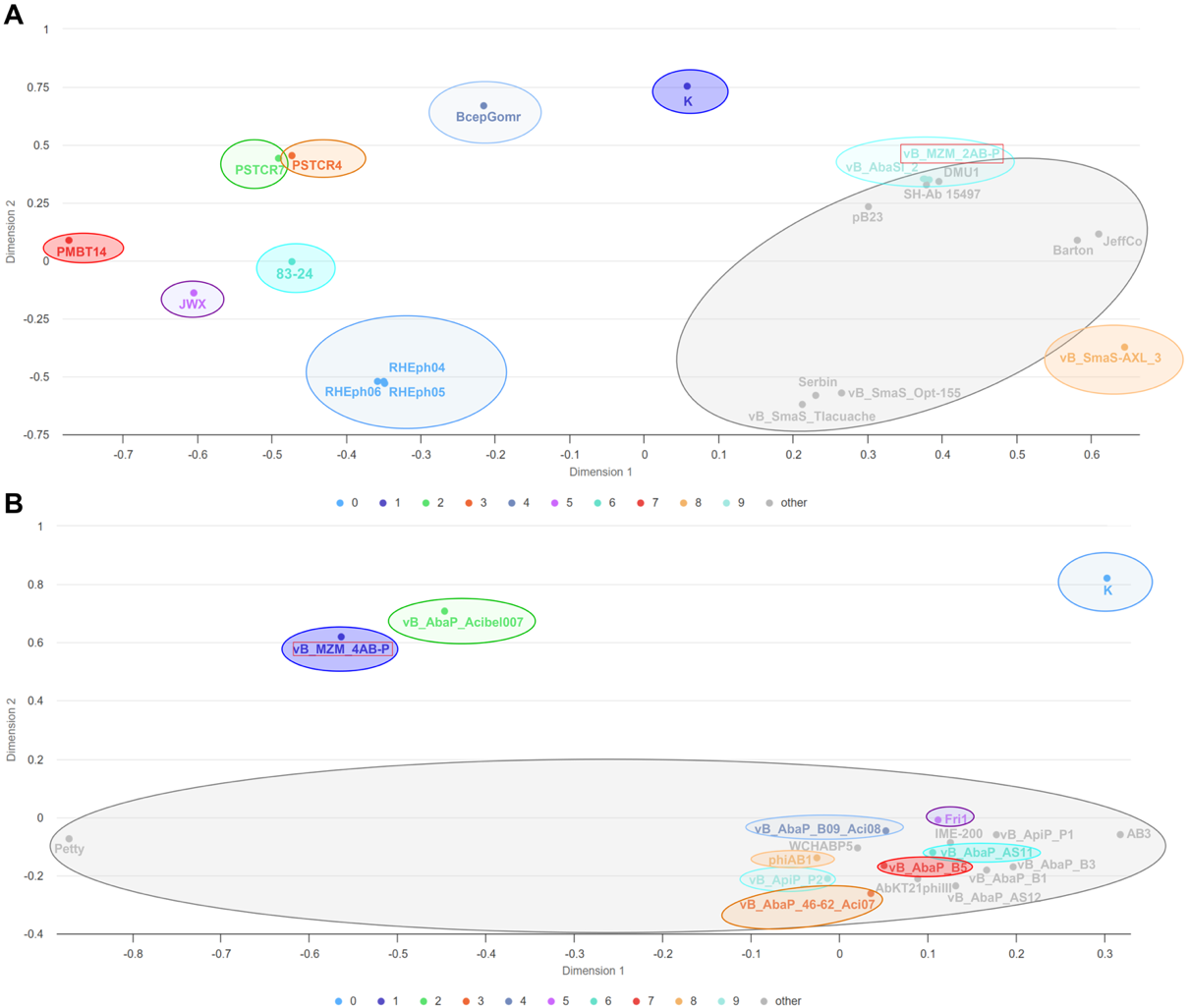
**

**Figure S3.** Dimensionality reduction by PCA of phage vB_MZM_2AB-P (A) and vB_MZM_4AB-P (B) of the sparse sequence similarity matrix clustered using Clusty. Sequence similarities were computed using k-mers (size=25) and a minimum k-mer count of 10. Clustering was performed with a minimum sequence identity threshold of 30%. Each point represents a viral genome, colored by its cluster assignment from the Leiden algorithm in Clusty.

**
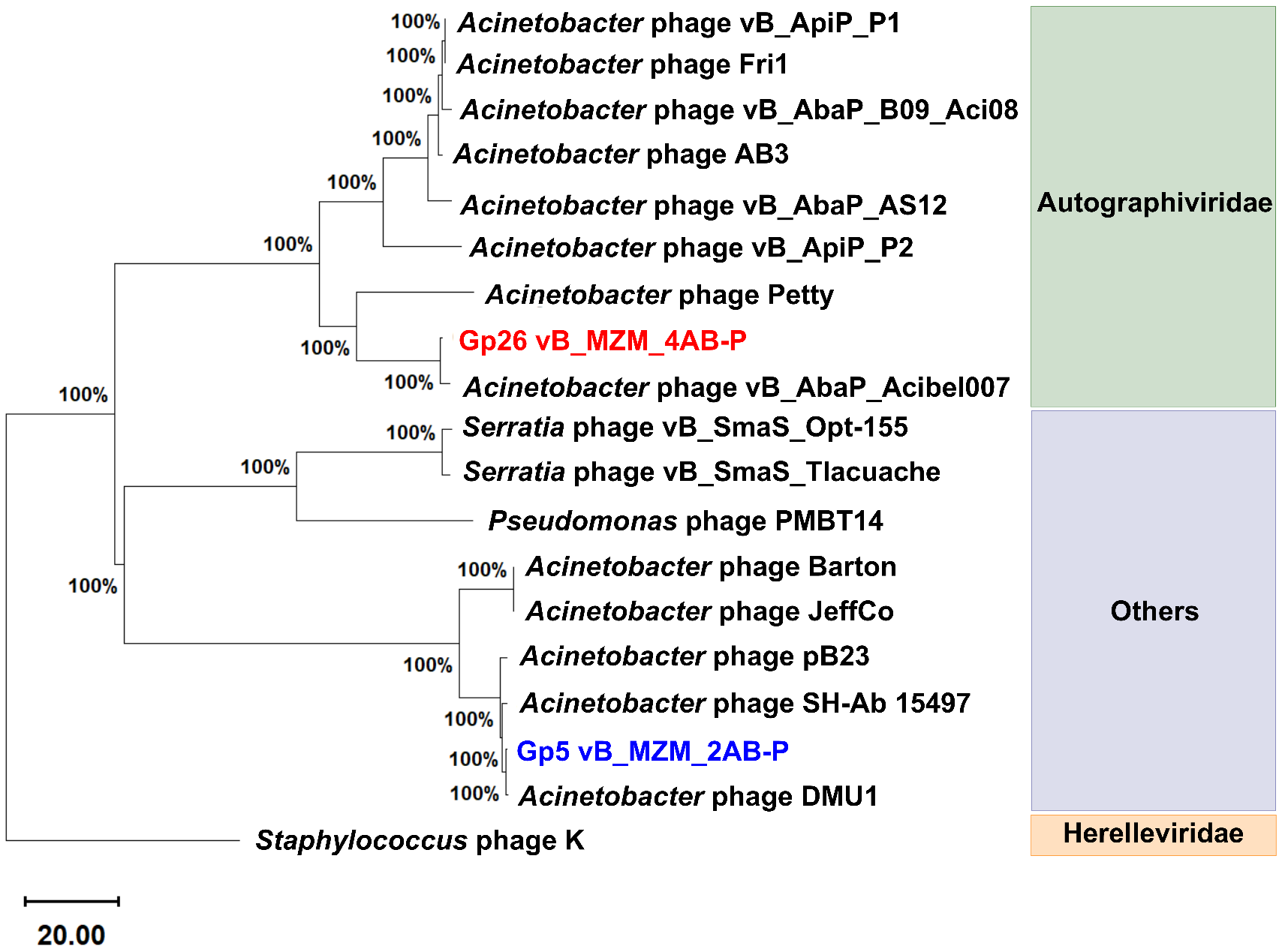
**

**Figure S4.** The phylogenetic tree was constructed based on the DNA polymerase (Gp5/Gp26) of phages vB_MZM_2AB-P and vB_MZM_4AB-P. The tree was generated using MEGA (version 11.0.11) with the neighbor-joining (N-J) method and a Bootstrap value of 1000. Phages marked with light green squares belong to the Autographiviridae family, those marked with light purple squares belong to Others, and phages marked with light orange squares belong to the Herelleviridae family. vB_MZM_2AB-P is marked in blue and vB_MZM_4AB-P is marked in red.

**Table S8.** Summary of biological characteristics of different *Acinetobacter* phages

| **Phage** | **Host** | **MOI** | **Latent phase (min)** | **Rise phase (min)** | **Burst size (PFU /cell)** | **Temperature stability** | **pH stability** | **Storage Stability** | **Reference** |
| --- | --- | --- | --- | --- | --- | --- | --- | --- | --- |
| vAbBal23 | *Acinetobacter* spp. | NA | 15 | NA | NA | NA | NA | NA | ^23^ |
| vAbAbd25 | *Acinetobacter* spp. | NA | 15 | NA | NA | NA | NA | NA | ^23^ |
| vB_AbaS_SA1 | *Acinetobacter* spp. | NA | 20-22 | 50 | 250 | NA | NA | NA | ^24^ |
| vB_AbaM_P1 | CGMCC 1.90331 | NA | 30 | 60 | 788 | NA | NA | NA | ^25^ |
| P1068 | *A.baumannii* ZWAb014 | NA | 10 | 60 | 280 | NA | NA | NA | ^19^ |
| Ab_WF01 | *Acinetobacter* spp. | NA | 10 | 40 | 151 | NA | NA | NA | ^26^ |
| pB23 | *A.baumannii* 2023 (B.m#2023) | NA | 20 | 60 | 217 | NA | NA | NA | ^27^ |
| vB_AbaM-SHI | *A.baumannii* SL | NA | 50 | 70 | 155 | NA | NA | NA | ^28^ |
| Scipio | *A.baumannii* LUH5534 | NA | 10 | NA | 45.5 ± 3.7 | NA | NA | NA | ^29^ |
| Brutus | *A.baumannii* MAR15-3273 | NA | 15 | NA | 75.0 ± 5.3 | NA | NA | NA | ^29^ |
| Phab24 | *A.baumannii* XH198 | NA | 30 | 140 | 350 | NA | NA | NA | ^30^ |
| vB_AbaM_ABPW7 | *A.baumannii* ABPW063 | NA | 20 |  | 145.25 ± 5.3 | NA | NA | NA | ^31^ |
| vB_AbaS_TCUP2199 | *A.baumannii* TV2199 | NA | 30 | 40 | 196 | NA |  | NA | ^32^ |
| VB_AbaM_AB4P2 | *A.baumannii* AB4 | 1 | 20 | NA | 61 | 30-70°C | 3-11 | NA | ^33^ |
| vB_AbaS_qsb1 | *A.baumannii* ioag01 | 0.01 | 10 | NA | 69 | 10°C- 60°C, but lost all activity at 80°C-100°C. | 3-11 | NA | ^34^ |
| vB_AbaP_ZC2 |  | 0.1 |  | NA |  | -80°C, 37°C, and 50°C, but was inactivated at 90°C. | 3-11 | -80°C | ^35^ |
| P425 | *A.baumannii* Ab25 | 0.00001 | 10 | NA | 184 | 4°C-50°C, but was completely inactivated at 60°C. | >70% within the pH range of 5- 11 | NA | ^36^ |
| Abgy202141 | *A.baumannii* GY-4 | 1 | 5 | NA | 189 | 4°C -55°C. | 4-12 | NA | ^37^ |
| vB_AbaSt_W16 | *A.baumannii* KBN10P02782 | 1 |  | NA |  | 4°C- 55°C, but nearly lost infectivity at 70°C. | 4-9 | NA | ^38^ |
| XC_1_ | *A. nosocomialis* | 0.01 | 20 | NA | 310 | 50°C and 60°C. | 7 | 4°C | ^39^ |
| TCUAN1 | NA | 10 | 30 | NA | 47 | NA | NA | NA | ^40^ |
| TCUAN2 | NA | 0.01 | 5 | NA | 64 | NA | NA | NA | ^40^ |
| HZY2308 | *A.baumannii* AB48 | 0.01 | 20 | NA | 4×10^6^ | 4°C-50°C, but lost all activity at 80°C. | 5-9 | NA | ^41^ |
| vB_AbaM_ABMM1 | *A.baumannii* TV2199 | 1; In vivo experiments in Zebrafish: 10 | 30 | NA | 284 | 4°C-37°C, but was completely inactivated at 60°C -70°C. | 5-9 | NA | ^42^ |
| vB_AbaAut_ChT04 |  | 0.001 | 10 | NA | 280 | 4°C- 37°C. | 5-8 | NA | ^43^ |
| MRABP9 | *A.baumannii* MRAB11 | NA | 10 | NA | 369 | -20°C, 4°C, 25°C, 37°C, 50°C, and 60°C, but was completely inactivated at 70°C. | 3-11 | NA | ^44^ |

"NA" represents for missing values.

**
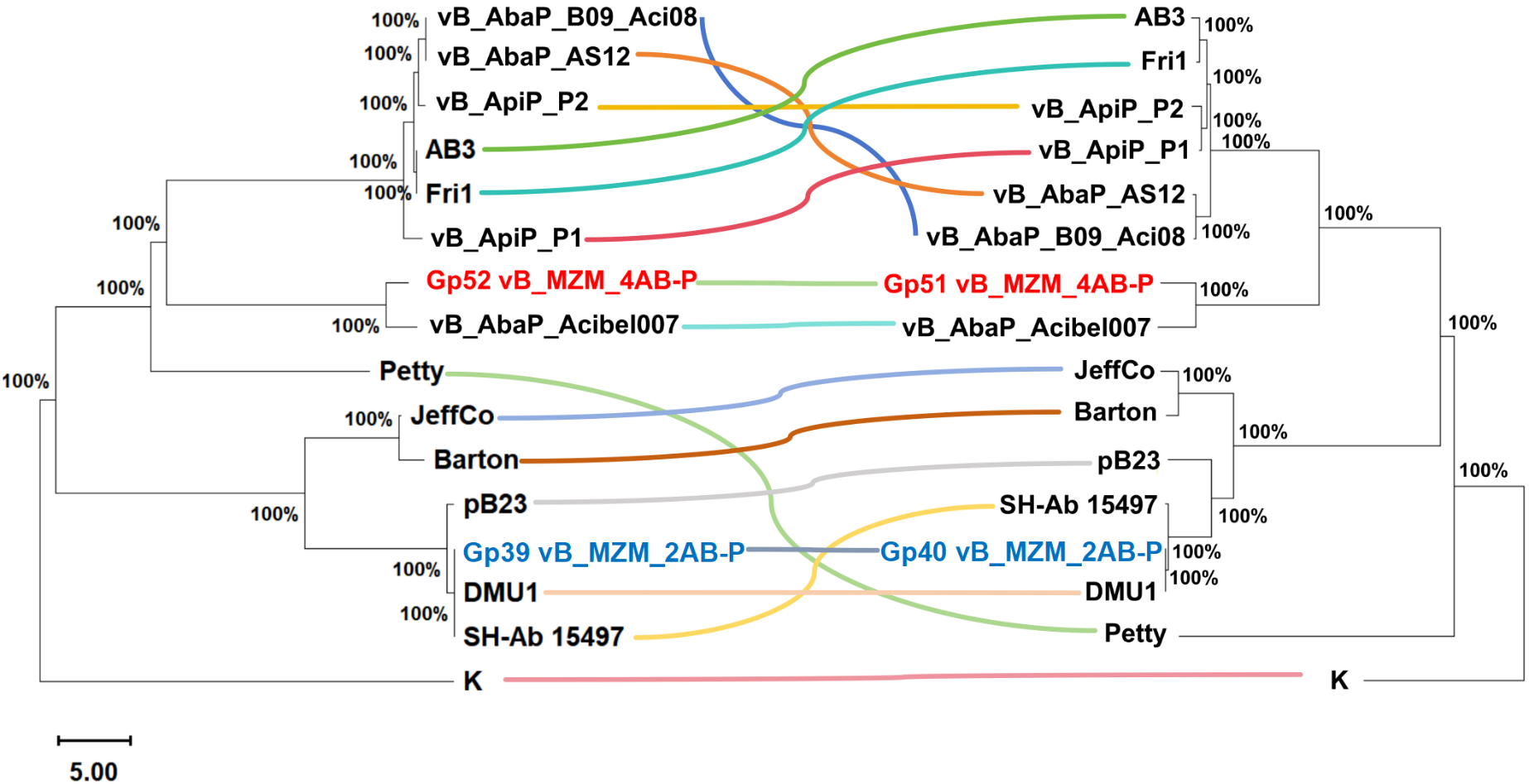
**

**Figure S5.** A phylogenetic tree was constructed based on holins (Gp39/Gp52) and endolysins (Gp40/Gp51) from vB_MZM_2AB-P and vB_MZM_4AB-P. This tree was generated using the Neighbor-Joining (N-J) method implemented in MEGA software (version 11.0.11), and the reliability of the tree branches was assessed through 1000 bootstrap replicates. Bootstrap values greater than 70% are indicated in the figure to ensure statistical significance. The figure highlights connections between holins and endolysins from the same phage by using identical-colored lines. Branches with different colors represent different phages, which facilitating differentiation. vB_MZM_2AB-P is marked in blue and vB_MZM_4AB-P is marked in red.


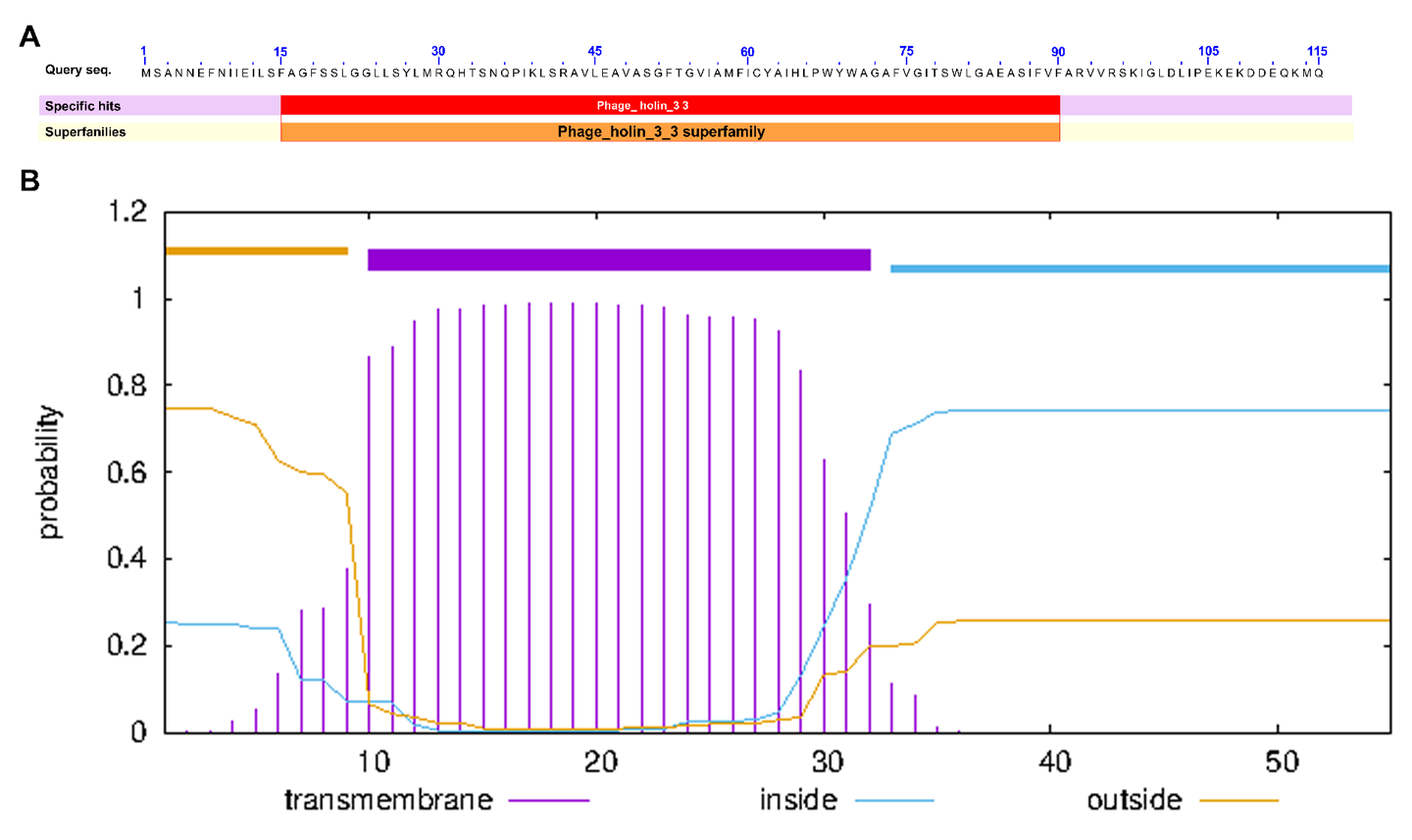


**Figure S6.** Domain prediction of Gp39 protein in phage vB_MZM_2AB-P. (A) CDD database analysis; (B) Transmembrane domain analysis.


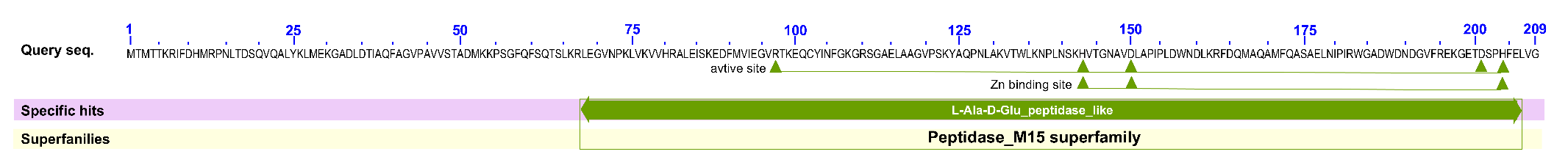


**Figure S7.** Domain prediction of Gp40 protein in phage vB_MZM_2AB-P based on CDD database.


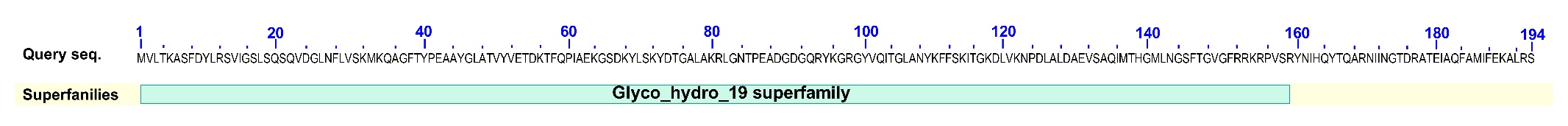


**Figure S8.** Domain prediction of Gp51 protein in phage vB_MZM_4AB-P based on CDD database.

**
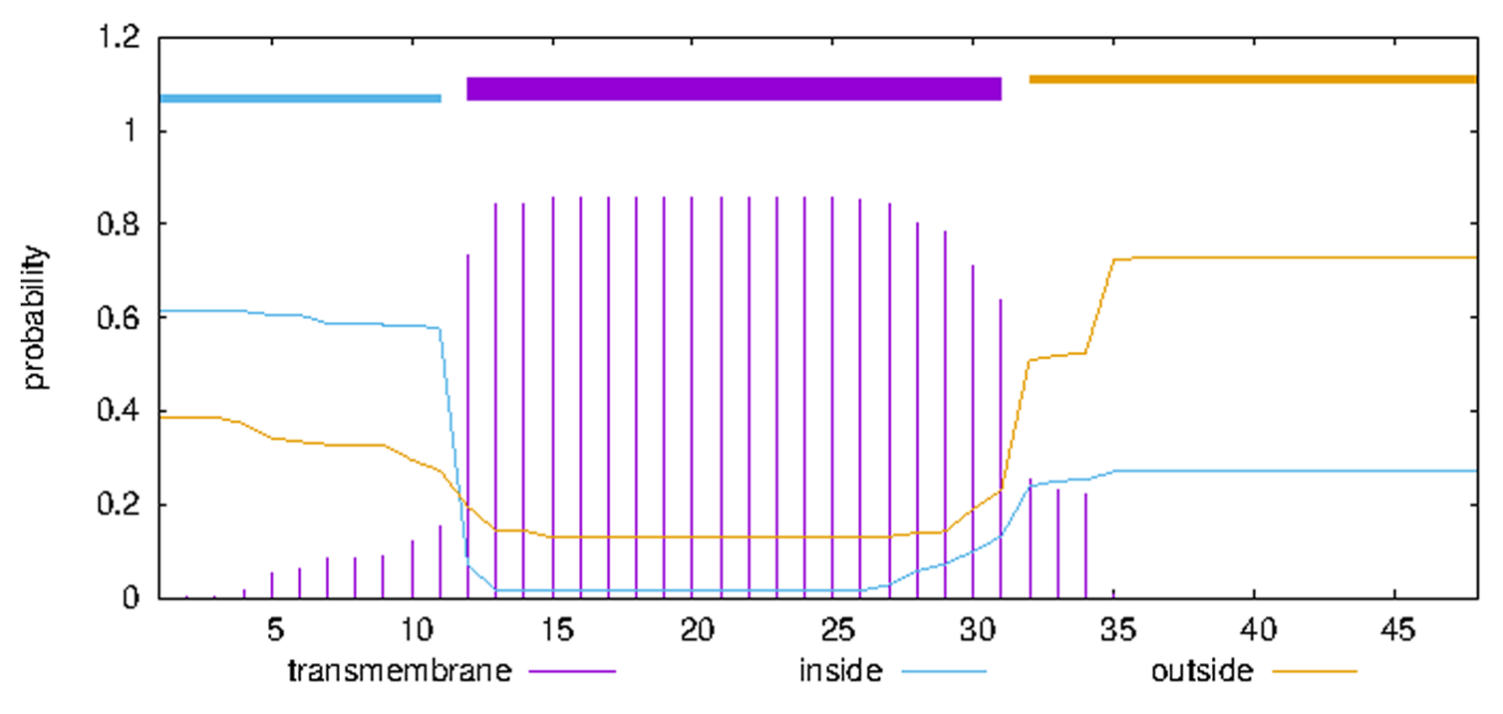
**

**Figure S9.** Transmembrane domain prediction of Gp52 protein in phage vB_MZM_4AB-P.

**Table S9.** Comparative analysis of phages vB_MZM_2AB-P and vB_MZM_4AB-P

| **Characteristic** |  | **Phage** |
| --- | --- | --- |
|  | **vB_MZM_2AB-P** | **vB_MZM_4AB-P** |
| Morphology | An icosahedral head with a diameter of 55.00 nm and a noncontractile tail length of 121.00 nm. | An icosahedral head with a diameter of 75.36 nm and a noncontractile tail length of 9.44 nm. |
| Host Range | *A. baumannii* 2AB, ATCC-19606 | *A. baumannii* 2AB, 4AB, 6AB |
| Adsorption Rate | 83.10% (5 min) | 85.00% (3 min) |
| Latent Period | 10 min | 10 min |
| Burst Size | 39.72 PFU/cell | 746.70 PFU/cell |
| Stability | Relatively stable at 60°C; relatively stable at pH 7-11; stored for 3 months at -20, 4, 28°C; generally stable. | Relatively stable at 50°C; relatively stable at pH 7; stored for 2 months at -20, 4, 28°C; generally stable. |
| Genome Features | linear dsDNA, 43664 bp | linear dsDNA, 42975 bp |

**References**

1 Peng, Q. *et al.* Characterization of bacteriophage vB_KleM_KB2 possessing high control ability to pathogenic *Klebsiella pneumoniae*. *Sci. Rep.* **13**, doi:10.1038/s41598-023-37065-5 (2023).

2 Kim, K. *et al.* H-NS is a transcriptional repressor of the CRISPR-Cas system in *Acinetobacter baumannii* ATCC 19606. *J Microbiol* **62**, 999-1012, doi:10.1007/s12275-024-00182-5 (2024).

3 Benyamini, P. The comparative characterization of a hypervirulent *Acinetobacter baumannii* bacteremia clinical isolate reveals a novel mechanism of pathogenesis. *Int. J. Mol. Sci.* **25**, doi:10.3390/ijms25189780 (2024).

4 Xiang, X. *et al.* Multiple mechanisms mediate aztreonam-avibactam resistance in *Klebsiella pneumoniae*: driven by KPC-2 and OmpK36 mutations. *Int. J. Antimicrob. Agents.* **65**, 107425, doi:10.1016/j.ijantimicag.2024.107425 (2025).

5 Bhushan, G. *et al.* Lipid A modification of colistin-resistant *Klebsiella pneumoniae* does not alter innate immune response in a mouse model of pneumonia. *Infect. Immun.* **92**, e0001624, doi:10.1128/iai.00016-24 (2024).

6 Peng, Q. *et al.* Isolation and characterization of a novel phage for controlling multidrug-resistant *Klebsiella pneumoniae*. *Microorganisms* **8**, 542 (2020).

7 Peng, Q. & Yuan, Y. Characterization of a newly isolated phage infecting pathogenic *Escherichia coli* and analysis of its mosaic structural genes. *Sci. Rep.* **8**, 8086, doi:10.1038/s41598-018-26004-4 (2018).

8 Hou, Y. *et al.* Establishment and application of a rapid visualization method for detecting *Vibrio parahaemolyticus* nucleic acid. *Infect. Med. (Beijing)* **3**, 100111, doi:10.1016/j.imj.2024.100111 (2024).

9 Wang, Y., Chen, K., Xing, Q., Zhang, T. & Xu, Y. Stachybotrins G and H, two new Phenylspirodrimane Derivatives from the fungus *Stachybotrys chartarum*. *Planta. Med.*, doi:10.1055/a-2592-1627 (2025).

10 Yao, X. *et al.* Synthesis, Antimicrobial activity, and molecular docking studies of Aminoguanidine Derivatives containing an Acylhydrazone Moiety. *Iran. J. Pharm. Res.* **20**, 536-545, doi:10.22037/ijpr.2020.113711.14446 (2021).

11 Bordel, S., Martín-González, D., Muñoz, R. & Santos-Beneit, F. Genome sequence analysis and characterization of *Bacillus altitudinis* B12, a polylactic acid- and keratin-degrading bacterium. *Mol. Genet. Genomics.* **298**, 389-398, doi:10.1007/s00438-022-01989-w (2023).

12 Pehde, B. M., Niewohner, D., Keomanivong, F. E. & Carruthers, M. D. Genome sequence and characterization of *Acinetobacter* phage DMU1. *Phage (New Rochelle)* **2**, 50-56, doi:10.1089/phage.2020.0043 (2021).

13 Zhou, Y. *et al.* A widespread pathway for substitution of adenine by diaminopurine in phage genomes. *Science* **372**, 512-516, doi:10.1126/science.abe4882 (2021).

14 Williams, E. A. *et al.* Complete genome sequence of *Serratia* marcescens Siphophage Serbin. *Microbiol. Resour. Announc.* **8**, doi:10.1128/mra.00422-19 (2019).

15 Dreiseikelmann, B. *et al.* Characterization and genome comparisons of three *Achromobacter* phages of the family Siphoviridae. *Arch. Virol.* **162**, 2191-2201, doi:10.1007/s00705-017-3347-8 (2017).

16 McCutcheon, J. G., Lin, A. & Dennis, J. J. Isolation and characterization of the novel bacteriophage AXL3 against *Stenotrophomonas maltophilia*. *Int. J. Mol. Sci.* **21**, doi:10.3390/ijms21176338 (2020).

17 Merabishvili, M. *et al.* Characterization of newly isolated lytic bacteriophages active against *Acinetobacter baumannii*. *PLoS One* **9**, e104853, doi:10.1371/journal.pone.0104853 (2014).

18 Popova, A. V. *et al.* Novel Fri1-like viruses infecting *Acinetobacter baumannii*-vB_AbaP_AS11 and vB_AbaP_AS12-characterization, comparative genomic analysis, and host-recognition strategy. *Viruses* **9**, doi:10.3390/v9070188 (2017).

19 Zheng, X. *et al.* Antibacterial activity evaluation of a novel K3-specific phage against *Acinetobacter baumannii* and evidence for receptor-binding domain transfer across morphologies. *Virol. Sin.* **39**, 767-781, doi:10.1016/j.virs.2024.08.002 (2024).

20 Huang, G. *et al.* Characterization and genome sequencing of phage Abp1, a new phiKMV-like virus infecting multidrug-resistant *Acinetobacter baumannii*. *Curr. Microbiol.* **66**, 535-543, doi:10.1007/s00284-013-0308-7 (2013).

21 Yuan, Y. *et al.* The endolysin of the *Acinetobacter baumannii* phage vB_AbaP_D2 shows broad antibacterial activity. *Microb. Biotechnol.* **14**, 403-418, doi:10.1111/1751-7915.13594 (2021).

22 Wu, M. *et al.* A novel phage PD-6A3, and its endolysin Ply6A3, with extended lytic activity against *Acinetobacter baumannii*. *Front. Microbiol.* **9**, 3302, doi:10.3389/fmicb.2018.03302 (2018).

23 Ndiaye, I. *et al.* Characterization of two *Friunavirus* phages and their inhibitory effects on biofilms of extremely drug resistant *Acinetobacter baumannii* in Dakar, Senegal. *BMC. Microbiol.* **24**, 449, doi:10.1186/s12866-024-03608-7 (2024).

24 Rastegar, S. *et al.* Characterization of bacteriophage vB_AbaS_SA1 and its synergistic effects with antibiotics against clinical multidrug-resistant *Acinetobacter baumannii* isolates. *Pathog. Dis.* **82**, doi:10.1093/femspd/ftae028 (2024).

25 Li, S. *et al.* A novel *Saclayvirus* *Acinetobacter baumannii* phage genomic analysis and effectiveness in preventing pneumonia. *Appl. Microbiol. Biotechnol.* **108**, 428, doi:10.1007/s00253-024-13208-0 (2024).

26 Wang, Z. *et al.* Characterization and efficacy against carbapenem-resistant *Acinetobacter baumannii* of a novel Friunavirus phage from sewage. *Front. Cell. Infect. Microbiol.* **14**, doi:10.3389/fcimb.2024.1382145 (2024).

27 Luo, J. *et al.* Synergy of lytic phage pB23 and meropenem combination against carbapenem-resistant *Acinetobacter baumannii*. *Antimicrob. Agents. Chemother.*, e0044824, doi:10.1128/aac.00448-24 (2024).

28 Jiang, L. *et al.* Characterization of a *Straboviridae* phage vB_AbaM-SHI and its inhibition effect on biofilms of *Acinetobacter baumannii*. *Front. Cell. Infect. Microbiol.* **14**, doi:10.3389/fcimb.2024.1351993 (2024).

29 Evseev, P. V. *et al.* New Obolenskvirus phages Brutus and Scipio: biology, evolution, and phage-host interaction. *Int. J. Mol. Sci.* **25**, doi:10.3390/ijms25042074 (2024).

30 Zhang, L. *et al.* Therapeutic evaluation of the *Acinetobacter baumannii* phage Phab24 for clinical use. *Virus Res.* **320**, 198889, doi:https://doi.org/10.1016/j.virusres.2022.198889 (2022).

31 Wintachai, P., Surachat, K., Chaimaha, G., Septama, A. W. & Smith, D. R. Isolation and characterization of a phapecoctavirus infecting multidrug-resistant *Acinetobacter baumannii* in A549 alveolar epithelial cells. *Viruses* **14**, doi:10.3390/v14112561 (2022).

32 Mardiana, M., Teh, S. H., Lin, L. C. & Lin, N. T. Isolation and characterization of a novel *Siphoviridae* phage, vB_AbaS_TCUP2199, infecting multidrug-resistant *Acinetobacter baumannii*. *Viruses* **14**, doi:10.3390/v14061240 (2022).

33 Su, J. *et al.* Characterization of a novel lytic phage vB_AbaM_AB4P2 encoding depolymerase and its application in eliminating biofilms formed by *Acinetobacter baumannii*. *BMC. Microbiol.* **25**, 123, doi:10.1186/s12866-025-03854-3 (2025).

34 Wang, J. *et al.* A novel genus of virulent phage targeting *Acinetobacter baumannii*: efficacy and safety in a murine model of pulmonary infection. *PLoS. Pathog.* **21**, e1013268, doi:10.1371/journal.ppat.1013268 (2025).

35 Essam, K. *et al.* Isolation and characterization of phages ΦZC2 and ΦZC3 against carbapenem-resistant *Acinetobacter baumannii*, and efficacy of ΦZC3 on A549 cells. *Virol. J.* **22**, 262, doi:10.1186/s12985-025-02885-6 (2025).

36 Lin, M. *et al.* Isolation and identification of a newly discovered broad-spectrum *Acinetobacter baumannii* phage and therapeutic validation against pan-resistant *Acinetobacter baumannii*. *Virol. Sin.* **40**, 587-600, doi:10.1016/j.virs.2025.06.003 (2025).

37 Tian, X. *et al.* Isolation, characterization and therapeutic evaluation of a new *Acinetobacter* virus Abgy202141 lysing *Acinetobacter baumannii*. *Front. Microbiol.* **15**, 1379400, doi:10.3389/fmicb.2024.1379400 (2024).

38 Choi, Y. J. *et al.* In vivo pharmacokinetics, therapeutic efficacy and immune response of bacteriophage vB_AbaSt_W16 against carbapenem-resistant *Acinetobacter baumannii*. *JAC. Antimicrob. Resist.* **7**, dlaf121, doi:10.1093/jacamr/dlaf121 (2025).

39 Wang, C. *et al.* Biological characteristics and genomic analysis of *Acinetobacter* nosocomialis lytic phage XC_1_. *Curr. Issues. Mol. Biol.* **47**, doi:10.3390/cimb47050335 (2025).

40 Pekkle Lam, H. Y. *et al.* Isolation and characterization of bacteriophages with activities against multi-drug-resistant *Acinetobacter* nosocomialis causing bloodstream infection in vivo. *J. Microbiol. Immunol. Infect.* **56**, 1026-1035, doi:10.1016/j.jmii.2023.07.012 (2023).

41 Wang, R. *et al.* Characterization of phage HZY2308 against *Acinetobacter baumannii* and identification of phage-resistant bacteria. *Virol. J.* **21**, 283, doi:10.1186/s12985-024-02556-y (2024).

42 Mardiana, M. *et al.* Characterization of a novel and active temperate phage vB_AbaM_ABMM1 with antibacterial activity against *Acinetobacter baumannii* infection. *Sci. Rep.* **13**, doi:10.1038/s41598-023-38453-7 (2023).

43 Leungtongkam, U. *et al.* Genome characterization of the novel lytic phage vB_AbaAut_ChT04 and the antimicrobial activity of its lysin peptide against *Acinetobacter baumannii* isolates from different time periods. *Arch. Virol.* **168**, 238, doi:10.1007/s00705-023-05862-y (2023).

44 Zhang, Y. *et al.* Characterization and therapeutic potential of MRABP9, a novel lytic bacteriophage infecting multidrug-resistant *Acinetobacter* *baumannii* clinical strains. *Virology* **595**, 110098, doi:10.1016/j.virol.2024.110098 (2024).
